# Carbon Source and Substrate Surface Affect Biofilm Formation by the Plant-Associated Bacterium *Pseudomonas donghuensis* P482

**DOI:** 10.3390/ijms25158351

**Published:** 2024-07-30

**Authors:** Magdalena Rajewska, Tomasz Maciąg, Magdalena Narajczyk, Sylwia Jafra

**Affiliations:** 1 Laboratory of Plant Microbiology, Intercollegiate Faculty of Biotechnology of University of Gdansk and Medical University of Gdansk, Abrahama 58, 80-307 Gdansk, Poland; sylwia.jafra@ug.edu.pl; 2 Institute of Biology, Department of Botany, Warsaw University of Life Sciences, Nowoursynowska 159, 02-776 Warsaw, Poland; tomasz_maciag@sggw.edu.pl; 3 Laboratory of Electron Microscopy, Faculty of Biology, University of Gdansk, Wita Stwosza 59, 80-308 Gdansk, Poland; magdalena.narajczyk@ug.edu.pl

**Keywords:** biofilm formation, nutrient source, pseudomonads, rhizosphere colonization, abiotic and biotic surface

## Abstract

The ability of bacteria to colonize diverse environmental niches is often linked to their competence in biofilm formation. It depends on the individual characteristics of a strain, the nature of the colonized surface (abiotic or biotic), or the availability of certain nutrients. *Pseudomonas donghuensis* P482 efficiently colonizes the rhizosphere of various plant hosts, but a connection between plant tissue colonization and the biofilm formation ability of this strain has not yet been established. We demonstrate here that the potential of P482 to form biofilms on abiotic surfaces and the structural characteristics of the biofilm are influenced by the carbon source available to the bacterium, with glycerol promoting the process. Also, the type of substratum, polystyrene or glass, impacts the ability of P482 to attach to the surface. Moreover, P482 mutants in genes associated with motility or chemotaxis, the synthesis of polysaccharides, and encoding proteases or regulatory factors, which affect biofilm formation on glass, were fully capable of colonizing the root tissue of both tomato and maize hosts. Investigating the role of cellular factors in biofilm formation using these plant-associated bacteria shows that the ability of bacteria to form biofilm on abiotic surfaces does not necessarily mirror its ability to colonize plant tissues. Our research provides a broader perspective on the adaptation of these bacteria to various environments.

## 1. Introduction

Biofilms are the prevalent form of bacterial growth in most ecosystems; it has been estimated that up to 80% of cells live in community-like clusters [1]. Bacterial cells forming biofilms may interact with biotic and abiotic surfaces, creating complex, multicellular structures which guarantee survival in specific environmental niches through protection from nutrient depletion, the activity of antibiotics and bactericides, harmful influences of the environment, predation, or physical damage [2,3,4,5]. Formed in various environments, biofilms exhibit different structures depending on the type of surface, physical conditions (e.g., temperature, humidity), nutrient availability, genetic background, and metabolic determinants of the bacteria [4,6,7,8].

Specific factors come into play when bacteria transition to a sessile lifestyle in biofilm, including those on cells’ surfaces. A crucial role is played by structures responsible for motility and efficient adhesion, such as flagella, type IV pili, or fimbriae [9,10]. It has been demonstrated for *Pseudomonas aeruginosa* and *P. fluorescens* that non-flagellated mutants are defective in biofilm formation both on biotic and abiotic surfaces [9,11]. However, some strains of *P. fluorescens* or *P. putida* do not require flagella to attach to abiotic surfaces if specific carbon sources (e.g., citrate) and ferric ions are present in the environment [9]. For other bacteria, such as *Bacillus cereus*, the presence of flagella decreased the ability of the cells to attach to a glass surface [12]. In contrast, in *Agrobacterium tumefaciens*, an aflagellate mutant formed aberrantly dense biofilms under flowing conditions [13]. The attachment abilities of bacteria are also expressed in the synthesis of exopolysaccharides, including Psl and Pel (major polysaccharides in *P. aeruginosa*), alginate, and lipopolysaccharides (LPS) [14,15]. The absence of the O-antigen component of the LPS in *P. aeruginosa* and *P. fluorescens* prevented the bacteria from colonizing abiotic surfaces or plant roots, respectively [16,17]. However, it has also been shown that the type of carbon source (e.g., glucose or citrate) influences the type IV pilus-dependent aggregation of bacteria [18,19].

Bacterial cells embedded in a biofilm matrix exhibit distinct metabolic characteristics, varying growth rates, and the differential expression and regulation of specific genes compared to their planktonic counterparts [3,20,21,22]. The extracellular matrix formed by polymers (EPSs, extracellular polymeric substances) composed of extracellular DNA (eDNA), proteins, and polysaccharides [23,24,25,26] protects the biofilm and serves as an architectural enhancer. Protein synthesis and the participation of ATP-dependent Clp proteases (i.e., ClpP) have also been shown to be required for biofilm formation [9]. The regulation of biofilm-related processes takes place at various levels in the bacterial cell, involving, among others, the Wsp (wrinkly spreader phenotype) or Pil-Chp (chemotaxis-related and sensory) systems [27], or the c-di-GMP second messenger, which is the highly conserved two-component regulatory system GacA/GacS (global activator of antibiotic and cyanide production) that also modulates the expression of genes involved in motility, the response to stress factors, and the synthesis of antimicrobial compounds and siderophores [28,29,30,31,32,33].

In the case of plant-associated bacteria, the formation of microcolonies on the host seeds, leaves, or roots is also considered biofilm development, which is exhibited by pathogenic, mutualistic, and neutral bacterial species [34,35,36,37,38] and may be the determinant of their competitive advantage in the environment [6]. Numerous plant growth-promoting rhizobacteria (PGPR) benefit from contact with host tissues and substances secreted by the plants, e.g., root exudates, in the presence of which they engage in chemotaxis [39]. Investigations of bacterial biofilm development on biotic and abiotic surfaces have revealed examples of the correlation between biofilm formation on artificial structures and bacterial adhesion to the surfaces of plant tissues, including roots in the rhizosphere [40,41,42]. For example, it has been shown for *P. fluorescens* that bacterial motility plays a more significant role in the colonization of roots [43]. On the other hand, in the case of *P. putida*, mutations in genes that encode adhesins negatively influenced the biofilm formation and bacterial attachment to biotic surfaces [41]. The rhizosphere-associated strain *P. putida* KT2440 was shown to attach to corn seeds through the putative surface and membrane proteins [44].

Much of the available data on biofilm formation and regulating factors are based on the well-described bacterial species, e.g., *P. aeruginosa*, *P. fluorescens*, or *P. putida*. How the process is influenced by environmental cues and what cellular components it involves in little-known species, such as *P. donghuensis* P482, requires investigation. P482 exhibits a not fully characterized antimicrobial activity towards plant pathogenic bacteria and fungi, the production of which is carbon source-dependent [45,46,47,48]. Genome sequencing did not reveal any of the typical elements involved in the synthesis of secondary metabolites of antimicrobial properties, known in pseudomonads. Also, no typical pseudomonads quorum sensing system such as *N*-acyl homoserine lactone (AHL)- or alkyl quinolone-dependent has been identified in P482. Atomic force microscopy observations have demonstrated that the P482 cells are flagellated at one of their poles (Supplementary Figure S1), enabling the bacterium to move in the environment and possibly participate in cell attachment to various surfaces. As a tomato rhizosphere isolate, P482 was demonstrated to successfully colonize the rhizosphere of plants [49] and exhibits adaptive responses to exudates from both tomato and maize [50]. To date, no published data are available on the determinants of biofilm formation on biotic or abiotic surfaces by *P. donghuensis* P482 or by any of its sibling strains (e.g., HYS^T^, SVBP6, P17) [51,52,53]. This prompted us to investigate the biofilm development of this bacterium from the point of view of both bacterial cell and environment-derived factors.

The analyses concerning plant-associated strains of bacteria focus on determining their ability to form biofilm in in vitro conditions, relating the results directly to interactions between plants and bacteria. The aim of this study was to compare, in vitro and in vivo, the mutants in genes related to biofilm formation in *P. donghuensis* P482 to verify whether the analyzed strains’ biofilm production in vitro reflects their ability to colonize plant tissues. Our findings, however, demonstrate that P482 strains deficient in biofilm formation on abiotic surfaces may still be capable of the efficient colonization of plant tissues (here, tomato and maize), in contrast to the usual predictions.

## 2. Results

Genome-wide analysis was performed to identify genes encoding cellular factors potentially involved in biofilm formation by *P. donghuensis* P482. Twenty knock-out mutants were obtained by allelic-exchange mutagenesis, selecting from the numerous genes identified in the genome based on the possible function comparison in other *Pseudomonas*. For the readers’ convenience, mutants are referred to in the text as gene names or abbreviations of gene products in the case of unannotated ones, as given in Table 1. To evaluate the impact of the introduced mutations on the motility of the P482 strains, swimming in semi-solid and liquid media and swarming assays were performed for all mutants. The analyses showed that, as could be expected, the motility of the bacteria was affected mainly by mutations in their flagellum synthesis genes (Supplementary Results, Supplementary Figures S2–S4).

### 2.1. EPS-Congo Red Binding Affected in P482 Mutants

**Figure 1:**
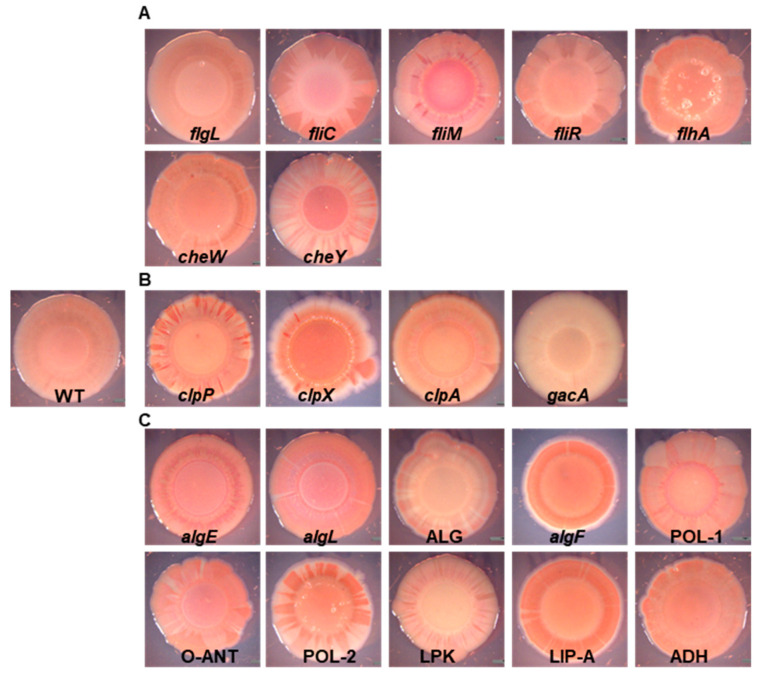
Colony morphology and Congo Red binding of the P482 wild-type and mutant strains. Images of Congo Red-stained colonies of the P482 WT and mutant strains: (**A**) knock-outs in motility and attachment-related genes, (**B**) knock-outs in proteases-encoding and *gacA* regulatory genes, (**C**) knock-outs in matrix synthesis-related genes.

The production of certain polymers which compose the matrix of biofilm, i.e., exopolysaccharides or lipopolysaccharides, allows for the auto-aggregation of cells and biofilm formation in bacteria, including plant-associated *Pseudomonas* [25,54,55,56]. One method for assessing the presence of EPSs is the colony Congo Red binding assay [24,57]. The P482 WT colony shows light pink pigmentation, with the dye evenly distributed in the bacterial mass (Figure 1), which might indicate relatively low levels of EPS in this experimental setup [57]. Two mutants showed no (*gacA*) or very reduced (LPK—lipopolysaccharide kinase family protein-encoding gene) Congo Red binding. The colony of the *gacA* mutant lacked any red pigment, possibly pointing to the regulation of the synthesis of the extracellular polymeric substances in P482 by the GacA/GacS system [58,59]. The LPK mutant, which had a lighter color than the wild type, is characterized by the deletion of a gene encoding a lipopolysaccharide kinase family protein. An interesting group of mutants was those where the colonies showed heterogenous pigmentation, with darker red sections in the center of the colony or dark-red stripes in the peripheral parts (Figure 1), such as those formed by *cheY* and *fliM* mutants (coding for a chemotaxis protein and the flagellar motor switch, respectively) (Figure 1A). A ‘striped’ colony phenotype can be observed for *clpP* and *clpX* mutants, in genes coding for ClpP and ClpX proteases, respectively (Figure 1B), and for strains with deletions in genes coding the O-antigen ligase family protein (O-ANT) and the polysaccharide biosynthesis/export family protein (POL-2) (Figure 1C). For two mutants, the *algF*, mutated in the gene coding for an alginate O-acetyl transferase, and LIP-A, carrying a mutation in a lipid-A-disaccharide synthase gene, a darker red color of the colony, with more dye bound to the matrix, was observed, hinting at higher levels of polysaccharide production in those mutants, and the possible use of pathways which do not involve activity of the two enzymes or divert sugar precursors to the synthesis of other polysaccharides.

### 2.2. Carbon Source Influences Biofilm Formation on Polystyrene

**Figure 2:**
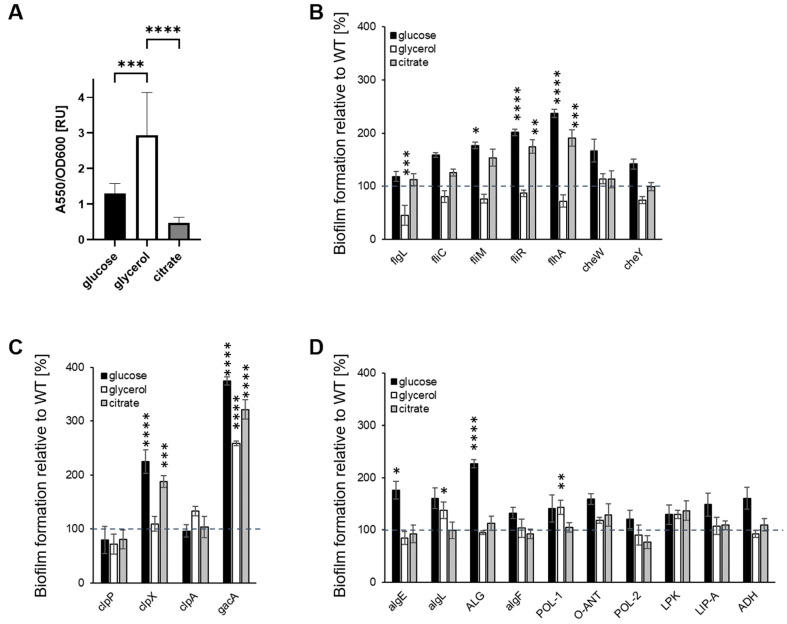
Biofilm formation by P482 mutant strains in crystal violet binding assay. Biofilm formation by P482 WT (**A**) and mutant strains, (**B**) knock-outs in motility and attachment-related genes, (**C**) knock-outs in proteases-encoding and *gacA* regulatory genes, and (**D**) knock-outs in matrix synthesis-related genes, on abiotic surface (polystyrene) in M9 minimal medium in relation to provided carbon source (22.2 mM glucose, 43.5 mM glycerol, 20 mM citrate). Biofilm formation by the mutant strains was determined by absorbance measurements normalized to those obtained for WT, taken as 100%. Grey dashed line marks 100% of biofilm formation by the WT strain. See Section 4 for details of the procedure. Absorbance was measured at 550 nm. The results represent mean values for three independent experiments with three sample replicates each. The error bars represent SEM. Statistical significance was determined with ANOVA and Dunnet’s multiple comparisons test for correction (* *p* < 0.05; ** *p* < 0.01; *** *p* < 0.0005; **** *p* < 0.0001); all *p* values for the ANOVA analyses can be found in Supplementary Tables S2 and S3.

First, the impact of the carbon source on the ability of the P482 WT to form biofilms was investigated. The biofilm formation was carbon source dependent: the biofilm biomass was most efficient when glycerol was present (Figure 2A), the production of biofilm was ca. two-fold lower with glucose, and the presence of citrate resulted in the lowest biofilm biomass.

Secondly, we sought to determine whether the carbon source influenced the biofilm formation in the P482 mutants when compared with the wild-type. None of the mutants showed a reduction in biofilm production when cultured with glucose (Figure 2B–D); three of the motility mutants (Figure 2B), *fliM*, *fliR*, and *flhA*, showed a significant increase (≥200%) in biofilm formation when compared to the wild-type strain, suggesting that, under these conditions, glucose may favor the transition of non-motile cells to surface-attached. Similar phenotypes were observed for the *clpX* protease mutant and the *gacA* mutant, with >2- and >3-fold increases in biofilm formation, respectively (Figure 2C). Also, two mutants, *algE* and ALG, in genes involved in the alginate biosynthesis, formed twice as much biofilm as the wild-type when grown with glucose (Figure 2D).

When the minimal medium was supplemented with glycerol as the sole carbon source, most of the mutants formed biofilms similar to that of the P482 WT, suggesting that the mutations did not affect the process. Only *flgL*, the mutant in a gene that codes for the flagellar hook-associated protein, demonstrated a >50% reduction in biofilm formation (Figure 2B). Three mutants showed an increased ability to form biofilms with glycerol as the sole carbon source. These were the *gacA* mutant, which produced an ~2.5-fold higher biofilm biomass than the wild-type, and two mutants associated with polysaccharide degradation and synthesis, *algL* (in a gene coding for alginate lyase) and POL-1 (in a gene coding for a polysaccharide biosynthesis protein) (Figure 2D). In the glycerol medium, the *P. donghuensis* P482 WT and mutants all exhibited much slower growth and a longer lag phase (see Supplementary Figure S5 and [47]) than in glucose or citrate containing media.

With citrate as a carbon source, four mutants showed increased biofilm formation compared to the wild-type; *fliR* and *flhA* both produced nearly twice as much (Figure 2B), while the *clpX* mutant produced three times more biofilm (Figure 2C). Increased biofilm formation was also observed for the *gacA* mutant (Figure 2C). All four mutants showed a similar biofilm phenotype to that exhibited in glucose medium. No differences in biofilm formation were observed compared with the wild-type strain for other mutants grown with citrate. Overall, the data show that biofilm formation by *P. donghuensis* P482 on the abiotic surface, polystyrene, is carbon source-dependent.

### 2.3. The Nature of the Abiotic Surface Impacts Biofilm Biomass and Architecture

To visualize the biofilm architectures of the P482 strains, confocal laser scanning microscopy (CLSM) was used to image the biofilms formed by each strain, which were fluorescently tagged with the pPROBE-GT-Kan plasmid, expressing green fluorescent protein (GFP), in glass-bottom 24-well plates.

**Figure 3:**
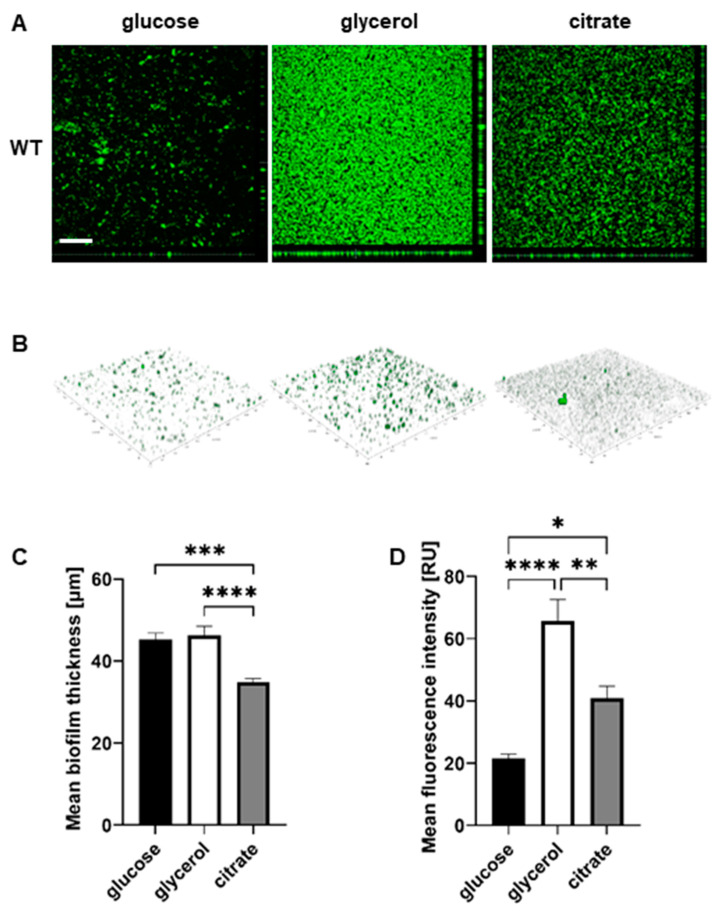
Biofilm formation by P482 wild-type strain on glass, in relation to carbon source. (**A**) Orthogonal and (**B**) 3D view of biofilm formed by GFP-tagged WT P482 strain on the glass bottoms of 24-well plates in M9 minimal medium supplemented with 22.2 mM glucose, 43.5 mM glycerol, or 20 mM citrate. (**C**) Measurement of mean thickness of the biofilm formed by the GFP P482 strain in the M9 medium with the given carbon sources. (**D**) Quantification of mean fluorescence intensity for the biofilm formed by the GFP P482 in the M9 medium with the given carbon sources. The bars represent means with SEM. Statistical significance was determined by one-way ANOVA (* *p* < 0.05; ** *p* < 0.01; *** *p* < 0.0005; **** *p* < 0.0001). Bar in (**A**) = 200 µm. Mean values of the biofilm thickness for P482 WT can be found in Supplementary Table S4.

The images obtained for P482 WT after growth in each of the three carbon sources revealed significant differences between the biofilm architectures on the glass surface. The bacteria formed a layer of cells in a glucose-supplemented medium, with single microcolonies scattered on the surface (Figure 3A,B). The biofilm architecture was much more developed for glycerol, with multiple microcolonies covering nearly the entire glass surface. In the presence of citrate, the biofilm formed by P482 WT exhibited a different phenotype when compared to those in glucose and glycerol—the cells were clustered into more microcolonies than were observed for glucose. Still, the biofilm was not as dense as in the case of glycerol (Figure 3A,B). Also, the biofilm in citrate was, relatively, the thinnest (mean 34.76 µm; with 44.95 and 46.58 µm in the cases of glucose and glycerol, respectively, see Supplementary Table S4) when *z*-stack measurements were considered (Figure 3C). The confocal microscopy images confirmed glycerol as the carbon source promoting the greatest biofilm biomass for *P. donghuensis* P482 when compared with those for glucose and citrate (Figure 3D). However, the biofilm formed on glass in the presence of citrate showed a larger biomass than the one in glucose, which contrasted with the results obtained for polystyrene.

**Figure 4:**
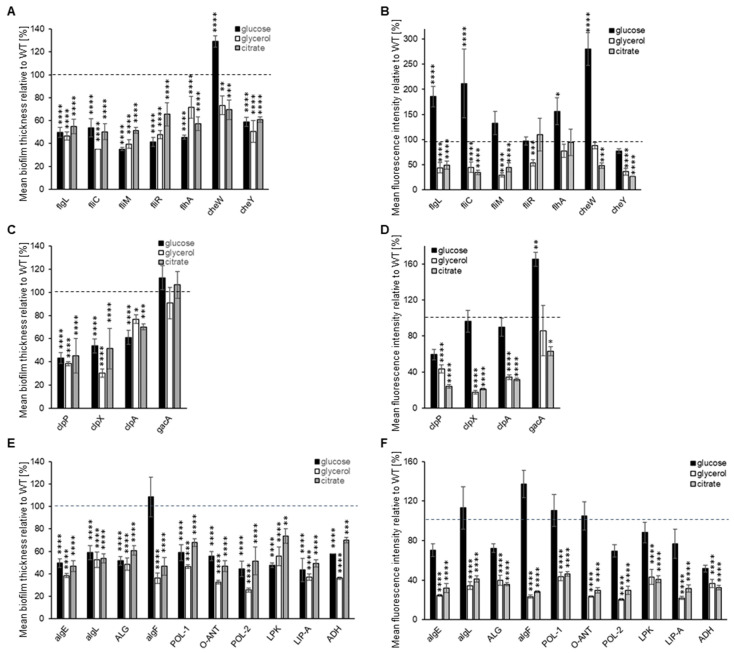
Biofilm formation on glass by GFP-tagged P482 mutants. The graphs (**A**–**C**) show measurements of biofilm thickness for all GFP P482 mutants, relative to WT, and (**D**–**F**) quantification of mean fluorescence intensity for the biofilm formed by the GFP P482 mutants, in the M9 medium supplemented with the individual carbon sources, done for *z*-stack images collected for (**A**,**D**) knock-outs in motility and attachment-related genes, (**B**,**E**) knock-outs in proteases-encoding and *gacA* genes, and (**C**,**F**) knock-outs in matrix synthesis-related genes. For reference, the 2D and 3D CLSM images can be found in Supplementary Figures S6–S8. Statistical significance for mutants vs. WT was determined with ANOVA and Dunnet’s multiple comparisons test for correction (* *p* < 0.05; ** *p* < 0.01; *** *p* < 0.0005; **** *p* < 0.0001); all *p* values for the ANOVA analyses can be found in Supplementary Tables S2 and S3. Mean values of the biofilm thickness for the mutant strains can be found in Supplementary Table S4.

The biofilm formation on glass was also evaluated for each of the P482 mutants. All mutants in flagellum-synthesis genes (*flgL*, *fliC*, *fliM*, *fliR*, and *flhA*) exhibited a diffused biofilm phenotype, regardless of the carbon source (Supplementary Figure S6). None of the five mutants formed clustered microcolonies in any of the tested media, with the most striking differences being observed for glycerol when compared with the wild-type strain. The biofilms formed in all three carbon sources were also thinner when compared to P482 WT (see Figure 4A and Supplementary Table S4). When taking into consideration the mean fluorescence reads, indirectly showing the amounts of cells engaged in the formation of the biofilm, the biomass of the biofilm was still noticeable in the case of the mutants *flgL*, *fliC*, and *flhA* when glucose was the carbon source (Figure 4B); however, with no visible microcolonies. This suggests that, in the presence of glucose, it might not be the flagella that determine the initial attachment to the surface. Still, they might be necessary for the active formation of the microcolonies. In glycerol and citrate, on the other hand, the *flgL*, *fliC*, *fliM*, and *cheY* mutants (the latter in a gene-encoding chemotaxis protein) demonstrated a significant decrease in biofilm formation, exhibited as single layers of cells attached to the surface. Interestingly, in glucose, *cheW*, the knock-out mutant in the chemotaxis protein CheW-encoding gene, formed a thicker (nearly 130%, relative to WT) biofilm with a much better-developed architecture than P482 WT (Figure 4A,B). In glycerol, it formed a thinner biofilm (ca. 70% of WT), although still with some distinguishable microcolonies, and in the presence of citrate, it formed a thin layer of cells with single microcolonies (Figure 4A,B and Supplementary Figure S6).

In the case of the *clpP* and *clpX* mutants, in all three tested carbon sources, a lack of microcolony formation was observed (Supplementary Figure S7), with a significant reduction in biofilm thickness and biomass in the presence of glycerol and citrate (Figure 4C,D, Supplementary Table S4). Glucose did not have that effect when it comes to biomass—the cells accumulated at the glass surface but in a diffuse manner (Supplementary Figure S7 and Figure 4D). This suggests that the activity of ClpP and ClpX proteases might be crucial for biofilm formation on glass when glycerol and citrate are present in the medium. Interestingly, the *clpA* mutant formed microcolonies in low-mass biofilm in the presence of citrate but very few in glucose or glycerol (Figure 4D and Supplementary Figure S7). The *gacA* mutant was able to form a clustered biofilm with microcolonies in a glycerol-containing medium (Supplementary Figure S7); in a glucose-containing medium, it produced more biomass than the P482 WT, with more microcolonies, but in a citrate-supplemented medium, its biofilm-forming ability was reduced to about 60% of the wild-type.

The mutations in exopolysaccharide and LPS synthesis-related genes all affected biofilm formation in glycerol and citrate in relation to P482 WT. The bacteria formed thin layers with very few, as in the case of *algF* or LIP-A mutants, or no microcolonies, as in the other matrix-related mutants (Figure 4D and Supplementary Figure S8). The presence of glucose again reduced the strains’ capacities to form complex biofilms. However, it facilitated biomass accumulation, as evidenced by the fluorescence signal intensity recorded in the analyzed z-stacks. (Figure 4E). This suggests that the capability of *P. donghuensis* P482 to develop a biofilm on a glass surface greatly depends on its ability to synthesize the polysaccharide components of the biofilm matrix, particularly when glycerol and citrate are the sole carbon sources. Overall, the analysis of the biofilm formation abilities of P482 on glass, as an abiotic surface, showed the complexity of the regulation of the process and suggested a possible role of the surface character of the substratum to which the cells attach.

### 2.4. P482 Biofilm-Defective Mutants Remain Capable of Colonizing Tomato and Maize Roots

To determine whether P482 mutants that were defective in biofilm formation on abiotic surfaces were also compromised in terms of plant root colonization, tomato and maize hosts were utilized to analyze the process for dicots and monocots, respectively.

**Figure 5:**
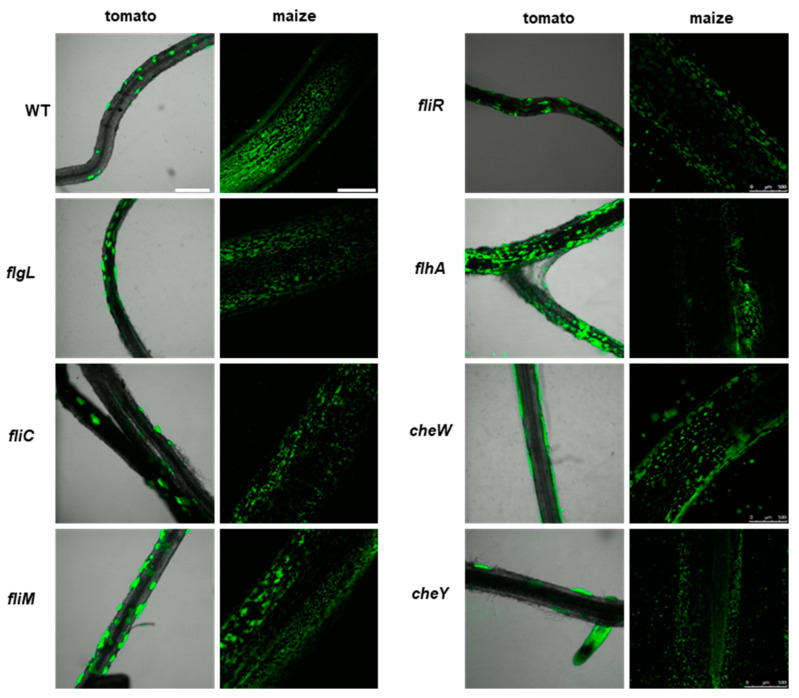
Colonization of plant rhizosphere by GFP-tagged P482 mutants in motility and attachment-related genes. Panels on the left show roots of tomato (merged bright-field and GFP channels), colonized with the P482 strains, visualized four weeks post-inoculation; panels on the right show P482-colonized maize roots (GFP channel), visualized one week post-inoculation (see Materials and Methods for details). Bars for tomato and maize = 500 µm.

The wild-type P482 strain colonized both types of plant rhizosphere efficiently, forming relatively large microcolonies scattered on the surface of tomato roots but more abundant fine colonies on the surface of maize roots (Figure 5, top left panel). The differences observed in the colonization patterns on the two plants might be due to the different culture conditions or the type of the root tissue (tomato vs. maize) itself. Nevertheless, in both experimental settings, P482 WT effectively colonized the root tissues and persisted on them for extended periods (one and four weeks, for maize and tomato, respectively). The motility mutants demonstrated a similar ability to attach to the roots of tomato and maize and formed clusters of cells, as observed for P482 WT (Figure 5), showing no defects in their capacities to form biofilm on the biotic surface. The *fliM* and *flhA* mutants appeared to form numerous, more densely packed microcolonies on tomato roots under the tested conditions when the strains were used individually as inoculants.

**Figure 6:**
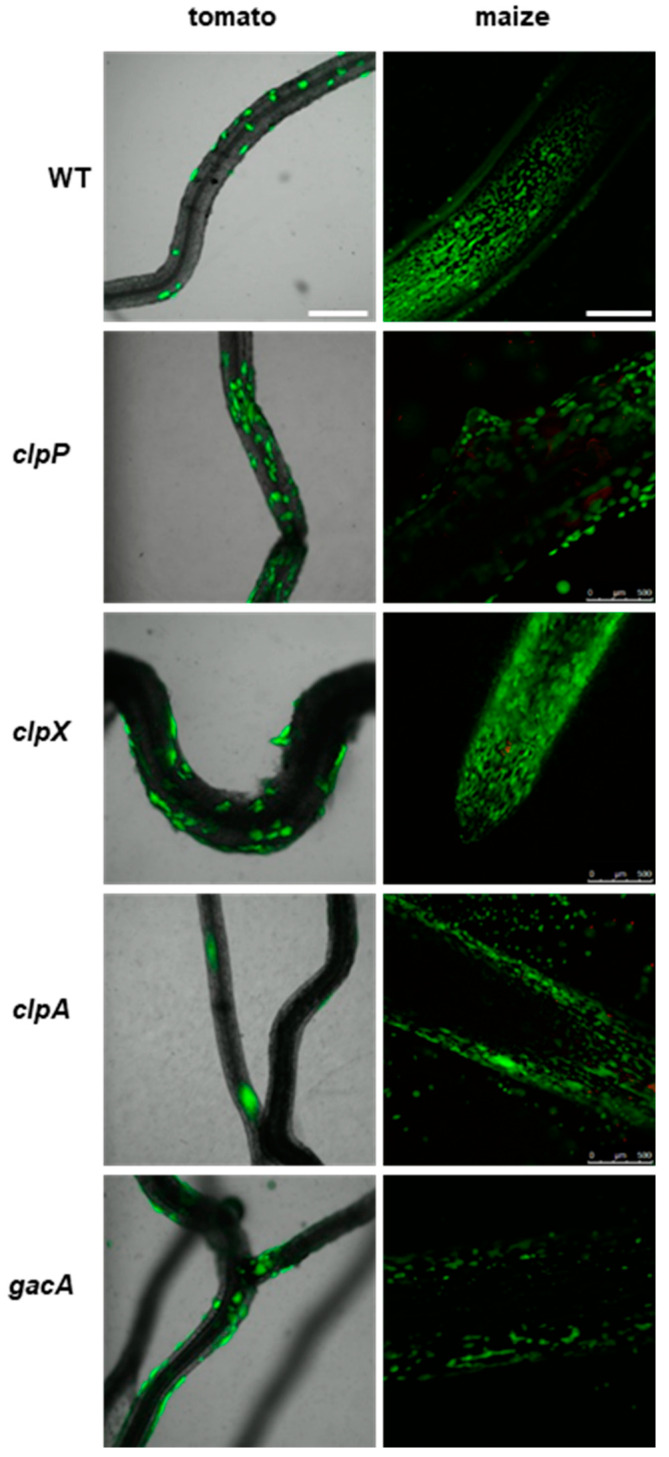
Colonization of plant rhizosphere by GFP-tagged P482 mutants in protease-encoding and *gacA* (KN3318) regulatory genes. Panels on the left show roots of tomato (merged bright-field and GFP channels) colonized with the P482 strains, visualized four weeks post-inoculation; panels on the right show P482-colonized maize roots (GFP channel), visualized one week post-inoculation (see Materials and Methods for details). Bars for tomato and maize = 500 µm.

The *clp* protease mutants exhibited similar patterns of colonization of the roots of tomato and maize to P482 WT (Figure 6). The *clpP* mutant appears to form slightly more microcolonies on the tomato roots than the wild-type strain. The *gacA* mutant showed no visible differences in tomato or maize rhizosphere colonization compared to P482 WT. This suggests that deletion of the *clp* proteases or *gacA* genes does not affect the biofilm formation on plant roots of P482.

**Figure 7:**
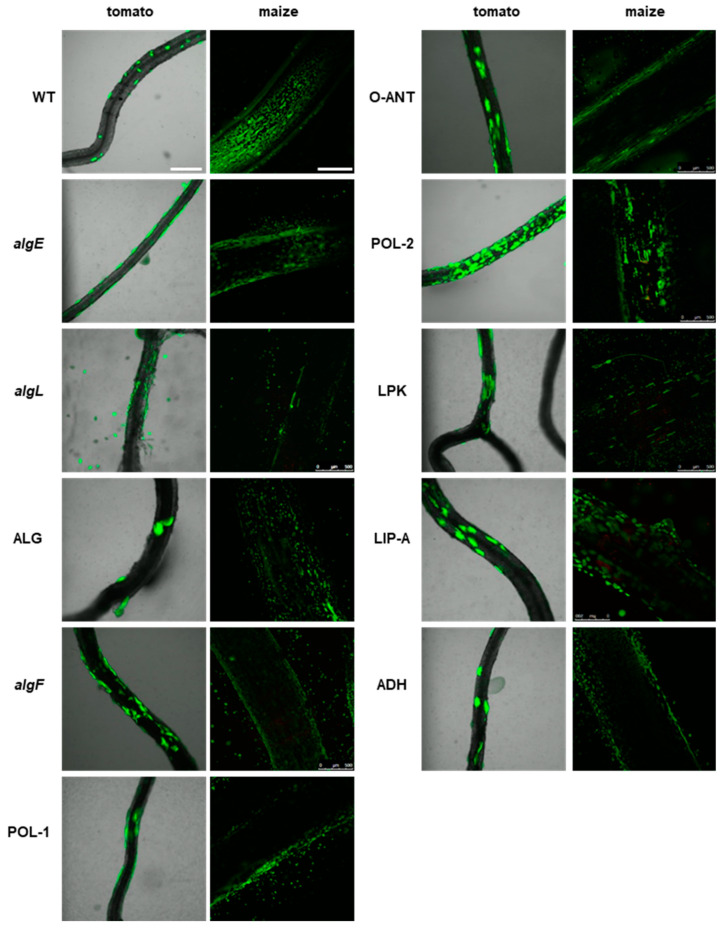
Colonization of plant rhizosphere by GFP-tagged P482 mutants in matrix synthesis-related genes. Panels on the left show roots of tomato (merged bright-field and GFP channels), colonized with the P482 strains, visualized four weeks post inoculation; panels on the right show P482-colonized maize roots (GFP channel), visualized one week post-inoculation (see Materials and Methods for details). Bars for tomato and maize = 500 µm.

Interestingly, among the mutants with deletions in genes associated with the synthesis of biofilm matrix components and adhesion, i.e., *algF*, O-ANT, and POL-2, are potentially hyper-colonizers of plant tissue, since the microcolonies were packed more densely on the surfaces of tomato roots (Figure 7). The remaining mutants in this group behaved similarly to the wild-type strain, forming scattered microcolonies on the tomato root tissue and multiple small colonies on the maize roots.

In summary, the mutations introduced in the P482 genome, associated with different functions in the biofilm formation process, did not decrease its plant root colonization abilities. The strains demonstrated different phenotypes when forming a biofilm on a biotic surface than those observed on abiotic surfaces, particularly glass, showing that the results obtained in vitro do not reflect those acquired in planta.

## 3. Discussion

The ability of plant-associated bacteria to form biofilms on the root tissues of their host is a trait enabling them to persist in the rhizosphere environment, giving them an advantage in the competition for nutrients [60,61,62,63]. Many PGPRs have been found to form biofilms on the roots of plants, and the process has been studied widely for well-established species of *Pseudomonas* (e.g., *P. fluorescens* F113, *P. putida* KT2440) [38,43,64,65,66,67]. However, the collected data do not allow us to unambiguously determine the components that are crucial for the formation of biofilms on plant tissues, and whether their regulation is species- or even strain-dependent. Data concerning newly established bacterial species are scarce and the factors behind the mechanisms of biofilm formation and plant tissue colonization are poorly understood. In this work, the influence of the carbon source and substrate surface (abiotic and biotic) on biofilm formation were studied for the plant-beneficial *P. donghuensis* P482 wild-type strain and a series of its isogenic mutants.

The transition from a planktonic to a sessile lifestyle is directly affected by the availability of nutrients in the environment [6,68], and carbon sources play possibly one of the leading roles in this process [69,70,71]. For the wild-type strain of *P. donghuensis* P482, differences in the utilization of glucose, glycerol, and citrate were reflected in the general production of biofilm biomass, as observed in the crystal violet assay, and in the biofilm architecture, visible in the CLSM images obtained for the GFP-tagged variant, as well as in growth rates of planktonic cells. Glycerol supplementation resulted in the most efficient biomass accumulation by P482 WT, even though it caused an extended lag phase [47]. In glycerol, the strain formed the most developed, structured biofilm, consisting of three-dimensional clusters of microcolonies. It was reported for *P. aeruginosa* that glycerol assimilation promotes biofilm formation in PAO1 to a greater extent than growth on glucose [72]. The mutation of *glpR*, which encodes the transcriptional repressor managing glycerol metabolism in *P. aeruginosa*, increased biofilm formation. Our work on the antimicrobial properties of the P482 showed that glycerol impacts the expression of genes involved in the synthesis of secondary metabolites when compared with glucose [47]. The possible role of glycerol and other carbon sources in regulating the transcription of biofilm-associated genes in P482 requires further investigation.

Many bacteria have evolved systems for the selection of carbon sources from the environment and the management of their utilization through complex regulatory pathways: carbon catabolite repression, control, and activation (CCR, CCC, and CCA, respectively) [73,74,75]. These contribute to optimizing bacterial metabolism when nutrients are limited, in the case of access to only one carbon source, when there is a need to utilize it in a balanced way to allow for the most efficient growth, or under stress conditions, also taking part in the regulation of quorum sensing systems and virulence [50,76,77,78]. Different carbon sources available to *P. aeruginosa* during the transition to a sessile lifestyle resulted in diverse biofilm architectures—glucose promoted the formation of a more structured, 3D biofilm [79,80]. Still, the presence of citrate caused it to remain mostly flat [10,18]. The P482 biofilm architecture did not reflect these observations. When grown with glucose, the biofilm formed by P482 WT was the least developed compared to that grown in glycerol; growth on citrate resulted in an intermediate type of biofilm. Glucose is not the preferred carbon source for *Pseudomonas* spp., although it is metabolized rapidly when present [75]. It did not cause a lag during the growth of P482 but negatively influenced the wild-type biofilm formation. It is plausible that, in sessile and planktonic P482 cells, different pathways for the given carbon sources are utilized and, in the transition phase, a metabolic switch occurs. Sessile *Clostridium thermocellum* cells exhibited an increased expression of genes involved in the catabolism of carbohydrates via glycolysis and pyruvate fermentation and functions critical for cell division, whereas the planktonic cells expressed motility and chemotaxis genes at higher levels [81]. What needs to be considered is that the P482 biofilm architecture might evolve depending on the maturity of the biofilm, as was shown for the *P. putida* strain grown in citrate [19]. Also, different mechanisms and/or regulation systems may be involved in P482 biofilm formation in other environmental conditions.

The P482 mutants demonstrated a range of biofilm phenotypes depending on the carbon source supplied and the nature of the colonized surface. For several motility and chemotaxis mutants (e.g., *fliR* or *flhA*), growth on glucose, on polystyrene or glass, resulted in a greater biofilm biomass than for P482 WT. In contrast, for glycerol or citrate, the same strains produced similar biofilms as the wild-type on polystyrene; however, on glass, almost all mutants showed a significant decrease in their ability to form biofilms and microcolonies or larger structures. Glucose may act as an inhibitor of flagellar motility, as was shown for *Vibrio vulnificus*, through the dephosphorylation of EIIA^Glc^ (a component of phosphoenolpyruvate: sugar phosphotransferase system, PTS) and sequestration of the FapA (flagellar assembly protein A) from the flagellated pole [82]. It was suggested as a mechanism allowing the bacteria to adapt to glucose-rich environments.

Moreover, initial biofilm formation in P482 might be mediated by structures other than flagella, but the accumulation of cells into clusters might depend on the flagella-based scaffolding of the biofilm [83]. For various strains of *P. aeruginosa*, it has been shown that their contact with the surface may also depend on the motility facilitated by the activity of type IV pili [9,84] or that neither of the organelles is required for attachment to the abiotic surface, as was shown for PAO1 [10,85]. The P482 genome contains numerous genes annotated as encoding type IV pilin proteins (e.g., *flp*), pilus assembly proteins (e.g., *pilX*), and type IV pili chemotaxis transducers. Therefore, it is worth investigating whether they might act as appendages, taking part in the early stages of biofilm formation and compensating for the absence of flagella.

The impact of nutrients and media supplementation with different carbon sources was analyzed for *P. fluorescens*, where glucose, citrate, or glutamate stimulated biofilm formation through partially intersecting pathways [9]. The effect of the carbon source was the more interesting, since some surface attachment-deficient mutants (i.e., non-motile strains and a *clpP* mutant) overcame the impact of mutation observed in glucose when supplemented with iron or grown in a minimal medium containing citrate or glutamate as the sole carbon source. In P482, a similar compensation mechanism could occur, since some mutants, biofilm-deficient in the presence of glycerol or citrate (relative to the wild-type strain), exhibited biofilm formation and biomass production with glucose in the medium.

The formation of biofilm is regulated by diverse regulatory mechanisms, including second messengers such as cyclic diguanosine-5′-monophosphate (c-di-GMP), small RNAs (sRNA), quorum sensing (QS) [86], and others. In *P. aeruginosa*, the GacA/GacS two-component system regulates the sRNAs, *rsmY* and *rsmZ*, which modulate the motility, secondary metabolite production, and exopolysaccharide synthesis through interactions with the sRNA-binding proteins RsmA and RsmN [28,29,30,87]. However, the effects of mutations within this regulatory system depend on the species involved. The increased expression of flagellin and enhanced motility have been observed in *gacA* mutants of root-colonizing *P. fluorescens* F113 [88]. In *P. aeruginosa* PAO1, the *gacA* mutant demonstrated increased swarming and biofilm formation [89]. In contrast, the P482 *gacA* mutant exhibited a non-motile phenotype in swarming assays and showed a reduced synthesis of matrix polysaccharides, as suggested by its colony Congo Red binding. However, the ability of the P482 *gacA* mutant to form biofilms on abiotic or biotic surfaces was mostly unaffected. Moreover, it produced ~3-fold more biofilm than the wild-type strain on polystyrene regardless of the carbon source utilized, indicating that biofilm formation is controlled negatively by this system in *P. donghuensis*. On the glass, the *gacA* mutant cells formed more microcolonies than the P482 WT when glucose was the sole carbon source but fewer when citrate was present. In contrast to glucose, we observed that the *gacA* expression in wild-type P482 was negatively affected in a medium supplemented with glycerol [47].

A possible pathway of biofilm regulation includes the activity of the Clp proteases, as shown for *P. fluorescens*, where a motile *clpP* mutant showed defects in biofilm formation on different types of abiotic surfaces [9]. The general function of proteases in bacterial cells is protein degradation, allowing for control of the cell physiology and stress responses; they consist of a peptidase subunit (ClpP) and an AAA+ unfoldase (ClpA or ClpX) [90]. For *P. aeruginosa*, it was demonstrated that isoforms of the ClpP peptidase play distinct roles in motility and biofilm formation, promoting the latter [91,92]. Also, in PAO1, the Lon protease was shown to be essential for biofilm formation [93], possibly through involvement in protein degradation, increasing the amino acid pool available during starvation, which occurs in the case of *E. coli* and *Salmonella typhimurium* [94]. The genes identified in *P. donghuensis* P482, encoding ClpP, ClpX, and ClpA, plausible subunits of an ATP-dependent Clp protease, are engaged in biofilm formation processes, as this activity of the *clp* mutants varied, depending on the surface and carbon source tested. Growth on glucose and citrate compensated for the *clpX* mutation on polystyrene, but on glass, citrate and glycerol negatively impacted the formation of biofilm. In *B. subtilis*, Clp proteases down-regulate the central metabolic pathways when cells enter a state of glucose starvation [95]. ClpXP also regulates the motility of this bacterium by controlling the levels of Spx, an oxidative and heat stress regulator which acts as a transient repressor of the transcription of flagellar genes [96]. These suggest the possible regulation of motility and, as a result, biofilm formation by proteases in P482, as shown for swarming defects in the *clpX* mutant.

Biofilm matrix EPSs synthesized by bacteria enable their survival and persistence through protection from external factors. Polysaccharide biosynthesis involves multiple energy-requiring processes and regulation [97,98] when different carbohydrates (mannose, glucose, rhamnose, mannuronic acid, etc.) are incorporated into the polymeric chains [99,100]. The *P. donghuensis* P482 genome encodes numerous enzymes involved in the synthesis of alginate, yet undefined exopolysaccharides, LPS, O-antigen, and enzymes catalyzing the cleavage reactions of the polymers. The phenotypic analyses utilizing the Congo Red staining of P482 colonies, where mutants (e.g., LPK, O-ANT, or POL-2) bound the dye less efficiently or the colony coloring was heterogenous, suggest either defective LPS production [101] or the possible local accumulation of matrix polymers due to the perturbed activity of enzymes involved in their synthesis and/or transport [102]. In our experimental setup, mutations in the genes encoding enzymes involved in the biosynthesis of matrix components in P482 had little impact on the biofilm formation on polystyrene, regardless of the carbon source utilized by the cells. Only in the case of two genes, encoding enzymes engaged in alginate synthesis (AlgE and alginate O-acetyltransferase), was increased biofilm production observed in the presence of glucose in relation to P482 WT. On glass, less biofilm formation was noted for all strains lacking the enzymes associated with matrix compounds’ biosynthesis in the presence of glycerol and citrate. However, the inability to develop microcolonies was observed for all three carbon sources.

Notwithstanding, the inactivation of enzymes which regulate the biosynthesis of polysaccharides mostly hinders efficient biofilm matrix production. It has been shown for *P. aeruginosa* PAO1 that glucose efficiently promotes biofilm formation by the upregulation of expression of the *pslA* gene, required for the biosynthesis of the Psl extracellular polysaccharide [103]. Mutation of the *glpR* regulator, which represses the expression of genes involved in glycerol metabolism, also enhances biofilm formation through the upregulation of the Pel polysaccharide [72]. However, in *P. aeruginosa* evolution experiments on *psl* and *pel* mutants, it was observed that the inactivation of *psl* leads to defects in initial biofilm formation but can promote the expression of compensatory matrix polysaccharides (*pel*) with a longer time of biofilm growth [104]. Extended biofilm growth experiments and gene expression analyses could shed more light on the nature and regulation of exopolysaccharide synthesis in the P482 biofilm matrix. Also, since the chemical composition and structure of matrix polysaccharides in P482 have not been studied, their isolation and characterization are underway.

The surface characteristics of the two different abiotic surfaces clearly impacted the interactions between P482 and the colonized environment. Physicochemical features of the artificial substrata, such as the surface charge, hydrophobicity, and roughness, but also the chemistry, topography, and conditioning greatly affect bacterial attachment [105,106,107,108]. The P482 mutants, irrespective of the type of introduced mutation, generally attach more efficiently to polystyrene than to glass, suggesting that specific surface sensing might play a role in biofilm formation by the bacterium. For *P. aeruginosa*, *E. coli*, and *Staphylococcus aureus*, it was demonstrated that the chemical character and specific coating of surfaces greatly affect biofilm formation [106]. The topography of polystyrene surfaces was shown to influence bacterial attachment, giving a possible surface design to prevent medical device-associated infections [107]. Borosilicate glass negatively affected the abilities of *P. aeruginosa* and *S. epidermidis* to form biofilms [109], but other polymers, in the case of *S. aureus*, *P. aeruginosa*, *E. coli* biofilms, also significantly reduced the cellular attachment and delayed it in time [110,111,112,113]. It has been widely suggested that the hydrophobicity of surfaces and bacterial cells, due to the composition of their cell wall molecules, e.g., proteins and lipids, and the character of the polymeric substances surrounding them in the biofilm matrix, are the traits promoting biofilm formation [79,113]. However, the processes involved in cellular attachment, bacteria–surface interactions, and sensing seem far more complex [114].

The ability of microbes to form biofilms on abiotic surfaces and the colonization of plant tissues do not simply go hand in hand and depend largely on the bacterial species. The complexity of bacterial adaptations to the sessile lifestyle is reflected in the variety of mechanisms that lead to the formation of biofilms [62,63]. In *P. putida*, the flagella and the systems of the synthesis and transport of large adhesin LapA play a role in attachment to both artificial surfaces and plant seeds, but the impairment of the synthesis of LPS was crucial only for seed and root colonization, not attachment to polystyrene [41]. In *P. fluorescens*, a surface attachment-defective mutant (*sad*) showed reduced biofilm formation on the abiotic surface but did not exhibit impairment in alfalfa root colonization [43]. For *P. donghuensis* P482, all investigated strains colonized the root tissues of both tomato and maize but showed different biofilm formation abilities depending on the abiotic surface, which indicates composite and alternative mechanisms governing these processes in this species. Also, the strains were analyzed individually in the colonization assays, and it would be interesting to evaluate their colonization abilities in competition tests, where the P482 WT would also be present. It was shown for *P. fluorescens* F113 that non-motile mutants that colonized alfalfa roots, when tested individually, were poor competitors when co-inoculated with the wild-type strain [115].

Moreover, the ability of P482 mutants to colonize plant roots can also be attributed to the biotic surface provided by the host. For various plant-associated bacteria, root exudates and substances secreted by the plant regulate the establishment of a microbiome in the host [38,62,116]. In recent work by Krzyżanowska and colleagues [50], P482 was shown to exhibit different patterns of gene expression in response to root exudates of the two plant hosts, tomato and maize. The induction of P482 genes associated with motility, e.g., *fimV*, involved in the assembly of type IV pili, or *fliS*, encoding one of the flagellar proteins, took place under the influence of maize exudates, in contrast to the reduction in the expression of genes associated with chemotaxis by tomato exudates. The plant root metabolites often serve as a trigger signal for the bacteria to colonize plant tissues and form microcolonies at sites where the exudates are released from cells to the soil [38,117], but can also act as biofilm formation inhibitors in relation to bacterial plant pathogens [118]. An essential trait in tomato root colonization by *P. fluorescens* WCS365 was the flagella-driven chemotaxis towards exudate components [119]. Also, the attachment of *P. fluorescens* F113 strains to alfalfa roots was possible, among other factors, due to a plant-derived mucigel that holds bacteria, forming microcolonies on the surface of the plant tissue [43].

## 4. Materials and Methods

### 4.1. Bacterial Strains and Culture Conditions

Bacterial strains, P482 wild-type (wt) and mutants (listed in Table 1), were cultured in Miller’s Lysogeny Broth (LB) or on LB solidified with 1.5% agar (Novagen, Merck Group, Darmstadt, Germany). When required, the media were supplemented with kanamycin (30 µg/mL) and/or gentamycin (40 µg/mL) for maintaining *P. donghuensis* P482 insertion mutants or GFP-tagged variants of the strains, respectively. For biofilm assays, the strains were cultured in a minimal M9 medium [120] supplemented with an appropriate carbon source. *P. donghuensis* P482 strains were grown at 28 °C, and *Escherichia coli* ST18 was grown at 37 °C in the media supplemented with 50 µg/mL of 5-aminolevulinic acid (5-ALA, Sigma-Aldrich, St. Louis, MO, USA) [121]. To determine bacterial growth rate and generation time, the cells were cultured in the minimal M9 medium supplemented with a carbon source in 96-well plates at 28 °C with shaking. The OD_600_ measurements were performed hourly using an EnVision Multilabel Reader (Perkin Elmer, Shelton, CT, USA) for 24 h.

### 4.2. Cell Micrography

Imaging of the P482 cells was performed using an atomic force microscope (AFM), as described in [122]. Bacteria were grown in the M9 minimal medium supplemented with 22 mM glucose overnight on a microscope glass cover slide placed in a Petri dish, with gentle shaking (60 rpm) at 28 °C. The slides were washed with water, and samples were fixed with 2.5% glutaraldehyde (Sigma Aldrich), washed, and dried in air. Cells were imaged in air using Bioscope Resolve (Bruker, Billerica, MA, USA), in ScanAsyst (Peak Force Tapping) mode, with the application of ScanAsyst Air probe (f0 7.0 kHz, diameter < 12 nm, k:0.4 N/m).

### 4.3. Insertion Mutagenesis

The P482 mutants analyzed in this study were obtained as previously described [45]. Briefly, fragments (392–624 bp) of selected genes (see Table 1) were amplified and cloned in the XbaI/XhoI, XbaI/KpnI, or XbaI/ApaI restriction sites of the pKNOCK-Km suicide vector [123]. The resulting constructs named pKN0543, pKN0864, pKN0868, pKN0887, pKN0892, pKN0894, pKN0850, pKN0883, pKN1101, pKN1102, pKN1251, pKN1850, pKN3824, pKN4694, pKN4697, pKN4698, pKN4700, pKN5088, pKN5092, and pKN5095 were introduced into the *E. coli* ST18 donor strain [121] and subsequently transferred to *P. donghuensis* P482 by biparental mating. The P482 transconjugants were screened for the presence of the pKNOCK-Km insertion with primers F_pKNOCK_backbone and R_pKNOCK_backbone (see [45] for details). The site of incorporation of the suicide vector into the target loci was confirmed through a sequencing reaction using the primer F_outof_pKNOCK [47] with the genomic DNA of each mutant as a template. This allowed for mapping the pKNOCK-Km insertion to single sites of the P482 genome for all mutants. The sequencing was performed at Oligo.pl (Warsaw, Poland). Sequences of the primer pairs used, annealing temperatures, and the expected lengths of amplicons are given in Supplementary Table S1. The targeted genes were divided into three subgroups (see Table 1): those predicted to be involved in motility or chemotaxis [124,125,126], those encoding proteases and the *gacA* regulatory gene [9,127], and those likely to be engaged in the synthesis of components of the biofilm matrix and/or attachment/adhesion [14,15].

### 4.4. Fluorescent Labelling of P482 Strains

GFP-expressing variants of the *P. donghuensis* P482 strains were obtained by transforming the P482 cells with pPROBE-GTkan vector [128] by electroporation (Gene Pulser Xcell; Bio-Rad, Hercules, CA, USA), as described in [49], with default pulse conditions set for *Pseudomonas* strains. The transformants were selected by plating the bacterial suspensions post-electroporation on LB-agar plates supplemented with kanamycin and gentamicin and verified for fluorescence in UV light.

### 4.5. Colony Morphology and Congo Red-Staining Assay

Colony morphology was assessed using a Congo Red assay as previously described [57]. Briefly, plates with M63 agar supplemented with 0.2% glucose, 0.5% casamino acids, 1 mM MgSO_4_, 1.5% agar, and 2 mL of a 50× Congo Red-Coomassie blue solution (2 mg/mL Congo Red and 1 mg/mL Coomassie blue) per 100 mL of medium were prepared. Three microliters of overnight cultures of each strain (ca. 4 McFarland units, McF) were applied on the Congo Red plates, which were incubated at 28 °C for 24 h and left at room temperature for 5 to 7 days for biofilm formation. Images of the colonies’ morphologies were taken with a Leica MZ10F stereomicroscope (Leica-Microsystems, Wetzlar, Germany).

### 4.6. Biofilm Formation Assays

#### 4.6.1. Crystal Violet Staining

The assay was performed on the polystyrene surface in 96-well microtiter plates, as previously described in [129], with modifications. Overnight cultures of the P482 WT strain and mutants were diluted to 1:100 in minimal M9 medium supplemented with 22.2 mM glucose, 43.5 mM glycerol, or 20 mM sodium citrate. Then, 150 µL aliquots were dispensed into a microtiter plate well (Sarstedt, Germany) in 4 replicates and incubated overnight at 28 °C without shaking. Following incubation, the plates were washed twice with distilled water to remove planktonic cells. The attached biofilm was stained using 150 µL of 0.1% crystal violet solution for 20 min and washed three times with distilled water to remove the excess stain. The biofilm-bound dye was re-solubilized by adding 150 µL of 30% acetic acid to each well. Absorbance at 550 nm was measured after 20 min incubation at room temperature using the Envision Multilabel plate reader. Biofilm formation was calculated by normalizing bacterial growth using OD_550/600_. The results obtained for the mutants were normalized to 100% of the absorbance measured for P482 WT. ANOVA analysis of variance was performed for the P482 WT and mutants with multiple comparisons. Dunnett’s or Tukey’s test was used to correct for multiple comparisons and determine differences between and within strains. The statistical analysis was done using GraphPad Prism 9.5.1 (GraphPad Software, Boston, MA, USA).

#### 4.6.2. Biofilm Formation on Glass

Biofilm formation on a glass surface was assessed in static conditions, as follows: overnight cultures of GFP-tagged *P. donghuensis* P482 WT and mutants were diluted to 1:500 in fresh M9 minimal medium supplemented with appropriate carbon source (22.2 mM glucose, 43.5 mM glycerol, or 20 mM sodium citrate), in glass-bottom 24-well microtiter plates (SensoPlate^TM^, Greiner Bio-One, Kremsmünster, Austria). The bacteria were incubated overnight at 28 °C without shaking; prior to imaging, the medium containing non-attached cells was removed, and the biofilms were covered with phosphate-buffered saline (PBS). Plates were analyzed with a scanning confocal microscope Leica SP8X (Leica-Microsystems, Wetzlar, Germany), using a 10× objective. Z-stack images were collected for every well, with an interval of 3.25 µm; the limits of stacks were set by moving within the *z*-axis range until no fluorescence was observed for both upper and lower stacks. The images were acquired and processed via Leica Application Suite X (LAS X 3.7.1.21655, Leica-Microsystems, Wetzlar, Germany) software, with four randomly selected fields of each sample. The 2D images were constructed from 3D *z*-stack images using the Maximum Intensity Projection algorithm in LAS X by selecting pixels of the highest intensity from every slice. Fluorescence data from the collected images were obtained by measuring the mean fluorescence intensity using the ImageJ 1.53t software. Biofilm thickness measurements were done with the LAS X software. ANOVA analysis of variance was performed for each of the analyzed P482 strains with multiple comparisons. Dunnett’s or Tukey’s test was used to correct for multiple comparisons to determine differences between and within strains. The statistical analysis was done using GraphPad Prism 9.5.1 (GraphPad Software, USA).

### 4.7. Tomato Rhizosphere Colonization

Tomato (*Solanum lycopersicum esculentum* L.) and maize (*Zea mays* L.) were chosen as representatives of the dicots and monocots, respectively, for the analyses concerning plant root colonization by *P. donghuensis* P482.

Tomato seeds (*S. lycopersicum* L., cv. Saint Pierre, Vilmorin Garden, Plewiska, Poland) were surface-sterilized by placing 20–30 seeds in a sterile Eppendorf tube and washing 3–4 times with sterile distilled water, then for 1 min in 70% ethanol, 3 min in 3% NaOCl, and then 3–4 times with sterile water again. The seeds were placed on 0.5× Murashige–Skoog agar plates and left to germinate in the dark for 4 days at 25 °C to select for those not contaminated with microorganisms. Subsequently, the seedlings were inoculated with suspensions of the GFP-tagged variants of the P482 WT and mutant strains in 10 mM MgCl_2_ at McF = 6. For every P482 strain, three germinated seeds were placed in a Petri dish, each covered with 10 µL of respective bacterial suspension and incubated at room temperature for 20 min. The inoculated seedlings were placed in Magenta boxes (Sigma-Aldrich, USA) containing 100 g sterile grit and 11 mL 1× Murashige–Skoog medium supplemented with 3% sucrose. Seedlings covered with MgCl_2_ served as a negative control. The boxes were incubated in a phytotron chamber for 4 weeks (16/8 h photoperiod, 20 °C). After incubation, root samples were prepared for microscopy, as described in [49], with modifications. Briefly, roots were cut and placed in petri dishes (6 cm diameter; Sarstedt, Germany), and covered with an enrichment medium (2 g/L KH_2_PO_4_, 4.66 g/L K_2_HPO_4_, 1.33 g/L (NH_4_)_2_SO_4_, 50 mg/L FeSO_4_, 80 mg/L MgSO_4_, 10 g/L agar, 0.4% glycerol, 40 mg/L gentamicin, 30 mg/L kanamycin). The samples were incubated overnight at 28 °C and analyzed with the Leica SP8X Confocal (Leica Microsystems, Germany), using a 10× objective and GFP excitation/detection settings (489/508 nm). Multiple images from different parts of the sampled roots were taken to evaluate tissue colonization by bacteria. The experimental setup was repeated two to four times for each P482 strain.

### 4.8. Maize Rhizosphere Colonization

Maize seeds (*Z. mays* L., cv. Bejm, Plant Breeding and Acclimatization Institute, Smolice, Poland) were surface-sterilized by immersion in 5% NaOCl for 15 min then in 70% ethanol for 10 min, and washed with sterile distilled water. The seeds were placed on water agar plates (15 g/L) and incubated in a phytotron chamber for 7 days for sprouting (16/8 h photoperiod, 20 °C). For inoculation of the maize sprouts, saline suspensions were prepared from the overnight cultures of GFP-tagged variants of P482 WT and mutant strains, McF = 1. Maize sprouts were immersed in the bacterial suspensions, two per strain, in petri dishes and incubated for 30 min at room temperature. Next, the sprouts were placed in sealed conical flasks containing 20 mL of sterile water and placed in the phytotron chamber for a further 7 days of growth. Sprouts immersed in clear saline served as a negative control. The preparation of root samples and the enrichment procedure were similar to those described for tomato colonization. The samples were incubated overnight at 28 °C and analyzed with a Leica HCS LSI Macro Confocal (Leica Microsystems, Germany), using a 5× objective and GFP excitation/detection settings. Multiple images from different parts of the sampled roots were taken to evaluate tissue colonization by bacteria. The entire experimental setup was repeated twice for each P482 derivative.

### 4.9. Motility Assays 

Swimming motility was determined as the diameter of the zone travelled by bacteria inoculated into 0.3% agar M8 plates, supplemented with 0.02% glucose, 0.05% casamino acids, and 1 mM MgSO_4_, and incubated for 18–20 h at 28°C ([130], modified). Swimming diameters were measured post-incubation using ImageJ. The data were analysed using GraphPad Prism 9.5.1 (GraphPad Software, USA). 

Swarming motility was assayed by applying bacteria onto the surface of 0.8% agar M8 plates supplemented with 0.1% glucose, 0.25% casamino acids, and 1 mM MgSO_4_ ([130], modified). Incubation and analysis of images were performed as described for the swimming assay. 

Bacterial motility in a liquid medium was determined using the motility medium S Base (HiMedia, Kennett Square, PA, USA), prepared according to the manufacturer’s protocol. The medium was inoculated using an inoculation loop with overnight bacterial cultures from LA plates. The tubes were incubated at 28 °C without shaking for 24 h; bacterial growth was observed and photographed. The assay was performed in three replicas.

## 5. Conclusions

The influence of mutations in selected *P. donghuensis* P482 genes, the carbon source, and the type of colonized surface (abiotic or biotic) on biofilm-related characteristics were investigated. The performed analyses showed that different carbon sources impact the ability of P482 to form a biofilm, with glycerol promoting the process. The investigation of P482 mutants revealed that mutants which are incapable of forming biofilm on glass surfaces demonstrated a contrasting capability to fully colonize plant root tissues. Additionally, we identified mutants that were unaffected in their biofilm formation, irrespective of the surface or carbon source used. These findings contribute to a deeper understanding of the subtle factors governing biofilm formation in P482, opening new opportunities to advance our comprehension of plant–microbe interactions.

## Figures and Tables

**Table 1:** Selected genes encoding proteins possibly involved in biofilm formation by *P. donghuensis* P482, targeted by site-directed mutagenesis.

Mutant’s Name	Locus	Annotated Gene	Product	Function in Biofilm Formation
*flgL*	BV82_0864	*flgL*	flagellar hook-associated protein 3	Motility/Chemotaxis
*fliC*	BV82_0868	*fliC*	bacterial flagellin *N*-terminal helical region family protein	
*fliM*	BV82_0887	*fliM*	flagellar motor switch protein FliM	
*fliR*	BV82_0892	*fliR*	flagellar biosynthetic protein FliR	
*flhA*	BV82_0894	*flhA*	flagellar biosynthesis protein FlhA	
*cheW*	BV82_0850	*cheW*	chemotaxis protein CheW	
*cheY*	BV82_0883	*cheY*	chemotaxis protein CheY	
*clpP*	BV82_1101	*clpP*	ATP-dependent Clp protease proteolytic subunit ClpP	Proteases/Regulatory
*clpX*	BV82_1102	*clpX*	ATP-dependent Clp protease ATP-binding subunit ClpX	
*clpA*	BV82_1251	*clpA*	ATP-dependent Clp protease ATP-binding subunit ClpA	
*gacA*	BV82_3318	*gacA*	GacA, a response regulator of the two-component GacS/GacA system	
*algE*	BV82_4694	*algE*	alginate production protein AlgE	Biofilm matrix/Adhesion
*algL*	BV82_4697	*algL*	alginate lyase	
ALG	BV82_4698	-	alginate O-acetyltransferase	
*algF*	BV82_4700	*algF*	alginate O-acetyltransferase AlgF	
POL-1	BV82_5088	-	polysaccharide biosynthesis family protein	
O-ANT	BV82_5092	-	O-antigen ligase family protein	
POL-2	BV82_5095	-	polysaccharide biosynthesis/export family protein	
LPS	BV82_1850	-	lipopolysaccharide kinase family protein	
LIP-A	BV82_0543	-	lipid-A-disaccharide synthase	
ADH	BV82_3824	-	adhesin	

## Data Availability

Requests for materials should be addressed to M.R.

## Supplementary Materials

The following supporting information can be downloaded at: https://www.mdpi.com/article/10.3390/ijms25158351/s1.
