# Supplementary material for: Carbon Source and Substrate Surface Affect Biofilm Formation by the Plant-Associated Bacterium Pseudomonas donghuensis P482
Source: Int J Mol Sci. 2024 Jul 30;25(15):8351. doi: 10.3390/ijms25158351 (PMC11312691; doi:10.3390/ijms25158351)
Supplement: Supplementary file 1 [file ijms-25-08351-s001.zip › ijms-3117132-supplementary.pdf]

SUPPLEMENTARY DATA

Carbon source and substrate surface affect biofilm formation by the plant-associated bacterium *Pseudomonas donghuensis* P482

Magdalena Rajewska[1,*], Tomasz Maciąg[2], Magdalena Narajczyk[3], Sylwia Jafra[1]

[1]Laboratory of Plant Microbiology, Intercollegiate Faculty of Biotechnology of UG and MUG, University of Gdansk, Abrahama 58, 80-307 Gdansk, Poland

[2]Institute of Biology, Department of Botany, Warsaw University of Life Sciences, Nowoursynowska 159, 02-776 Warsaw, Poland

[3]Laboratory of Electron Microscopy, Faculty of Biology, University of Gdansk, Wita Stwosza 59, 80-308 Gdansk, Poland

*** Correspondence:** magdalena.rajewska@biotech.ug.edu.pl

Supplementary Results

Motility of the *P. donghuensis* P482 cells is dependent on the activity of flagella

To assess whether the introduced mutations had any effect on the motility of the P482 strain, a step of the biofilm formation process, swimming in semi-solid and liquid media, and swarming assays were performed for all mutant strains (listed in Table 1). The swimming analyses showed that, as it could be expected, the mutants affected in motility were those where flagellum synthesis was disturbed. All five flagellum-synthesis related mutants, in the *flgL* gene encoding the flagellar hook-associated protein, *fliC* encoding flagellin, being the main component of the filament, *fliM* – coding for the motor switch protein, which allows for motion and rotation of the flagellum, and *fliR* and *flhA* encoding proteins involved in flagellar export, showed defects in the swimming motility when compared to P482 wt (Supplementary Fig. S2 A-C). Similar result was obtained for the swarming motility. The five mutants in flagellum-related genes were unable to swarm (Supplementary Fig. S3 A-B), as compared to the P482 wt, and the growth of the mutants was visible only at the inoculation spot. The only other mutant that exhibited a significant defect in the swarming motility was the *gacA* mutant. Other mutants exhibiting a negative impact on swarming were the *clpX* mutant encoding one of the intracellular family of Clp proteases and the POL-1, which carries a deletion in a gene predicted to be involved in polysaccharide biosynthesis (see Supplementary Fig. S3A and B; P values > 0.05). In contrast, the mutant in the *cheW*, a chemotaxis protein-encoding gene, was a hyperswarmer (Supplementary Fig. S3A and B). These findings demonstrate that akin to other *Pseudomonas*, swarming in *P. donghuensis* relies on the flagella. Additionally, the involvement of the *gacA* response regulator gene, along with other factors, appears to be pivotal in influencing this activity. Electron microscopy imaging revealed that the *gacA* mutant does not possess flagella (Supplementary Figure S4), implying this regulator's involvement in the synthesis of these organelles in P482. Overall, the results suggest that *P. donghuensis* P482 motility depends on the active flagella in the case of swimming and swarming.

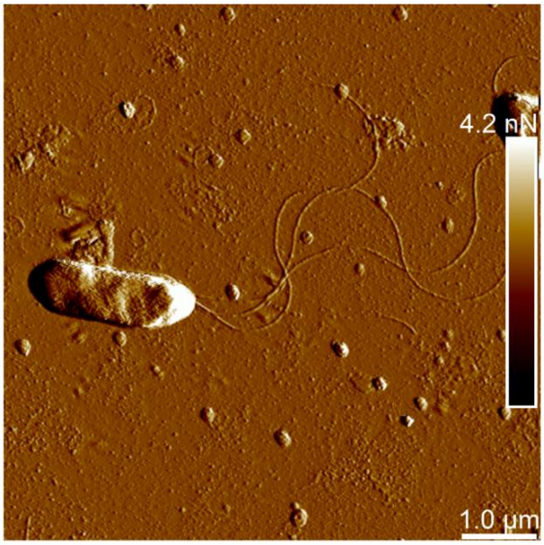

Supplementary Figure S1. AFM micrograph of the *P. donghuensis* P482 wild-type. Imaging was performed in air using Bioscope Resolve (Bruker), in ScanAsyst (Peak Force Tapping) mode, with the application of ScanAsyst Air probe (f_0 7.0 kHz, diameter <12 nm, k: 0.4 N/m).

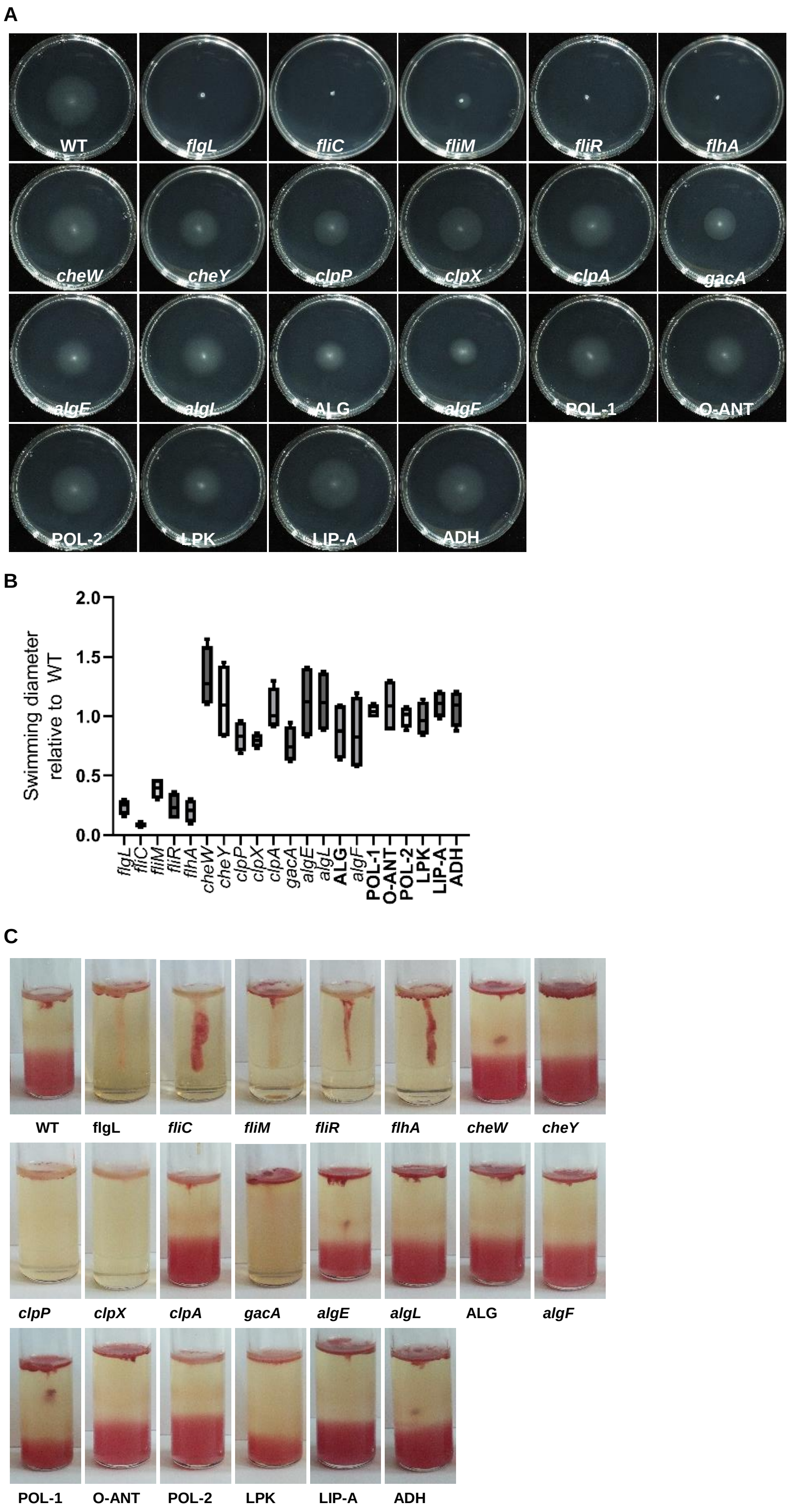

Supplementary Figure S2. Swimming motility data for all analysed P482 strains. (A) Representative images of all analysed P482 mutants versus the wild-type strain. (B) Quantification of swimming diameter of all analysed mutants relative to the wild-type strain (taken as 1), n=4. (C) Results of motility assay in motility S medium for all analysed P482 mutants.

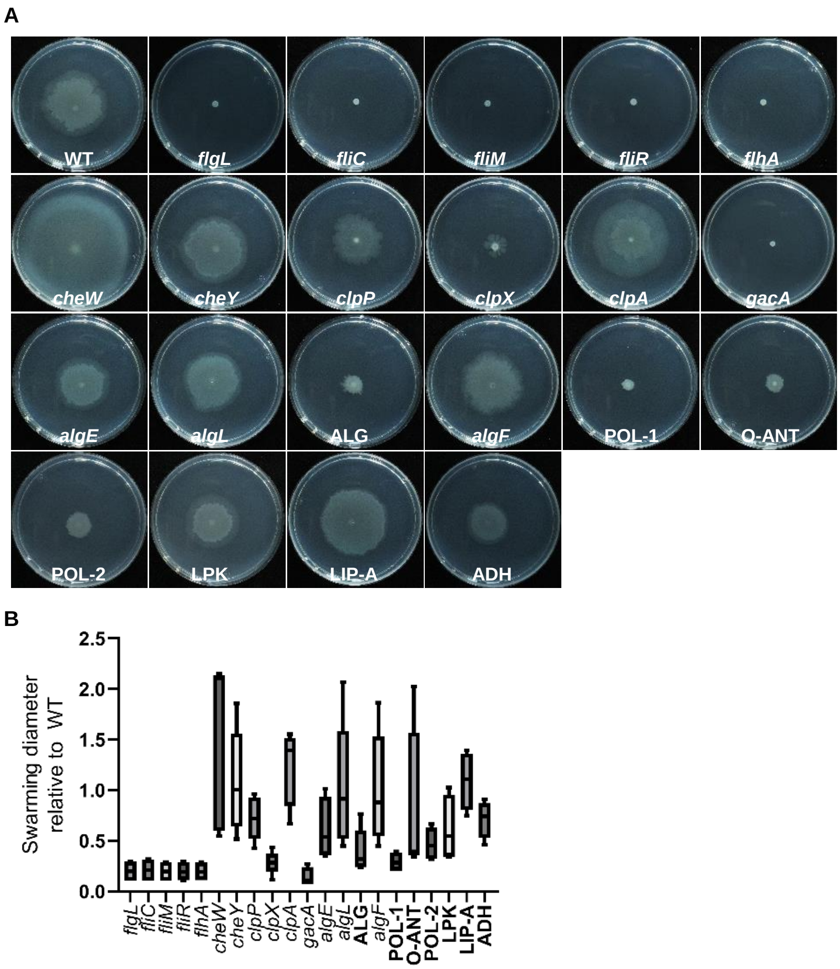

Supplementary Figure S3. Swarming motility data for all analysed P482 strains. (A) Representative images of all analysed P482 mutants versus the wild-type strain. (B) Quantification of swarming diameter of all analysed mutants relative to the wild-type strain, n=4 or 5.

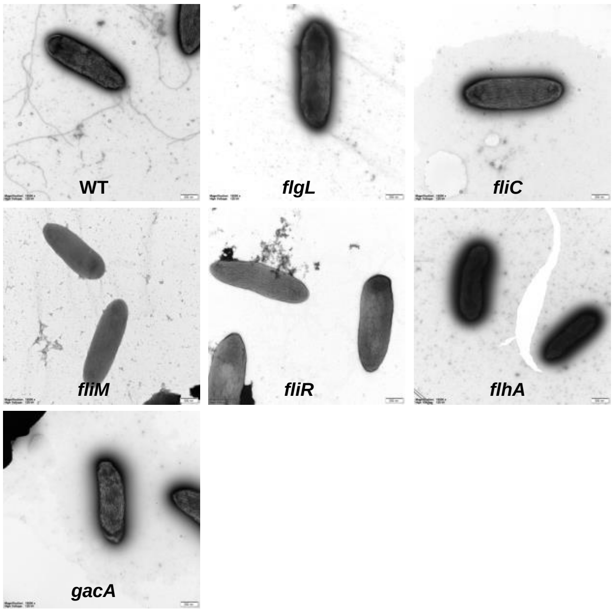

Supplementary Figure S4. TEM micrographs of the *P. donghuensis* P482 wild-type and non-motile mutant strains. Imaging was performed with a transmission electron microscope, for PBS-washed cells adsorbed on carbon-coated grids, stained with 1.5% uranyl acetate.

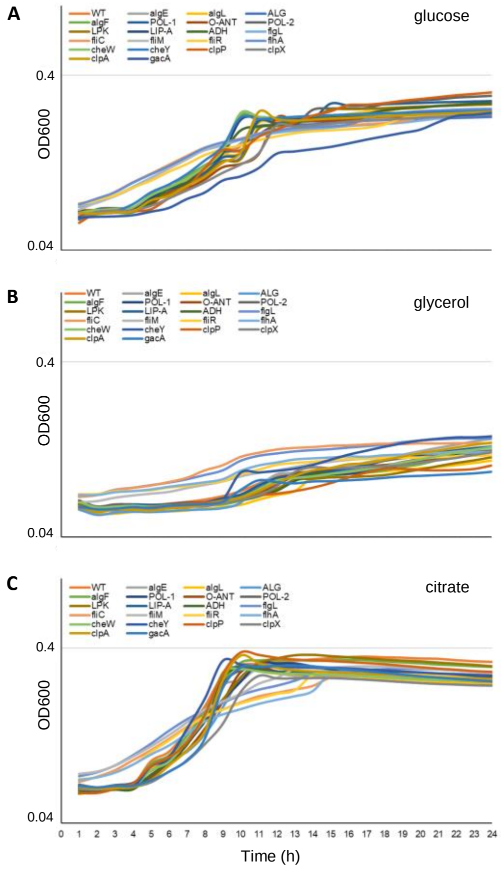

Supplementary Figure S5. Growth curves for *P. donghuensis* P482 wild type strain and its mutants in minimal medium supplemented with different carbon sources. The strains were cultured in M9 medium supplemented with (A) 22.2 mM glucose, (B) 43.5 mM glycerol or (C) 20 mM citrate, in 96-well plate in 3 technical replicates each, for 24 h at 28°C, with shaking. The 600 nm absorbance measurements (OD_{600}) were done every 20 minutes. Mean value for each readout was used to produce growth curves of each strain under given conditions.

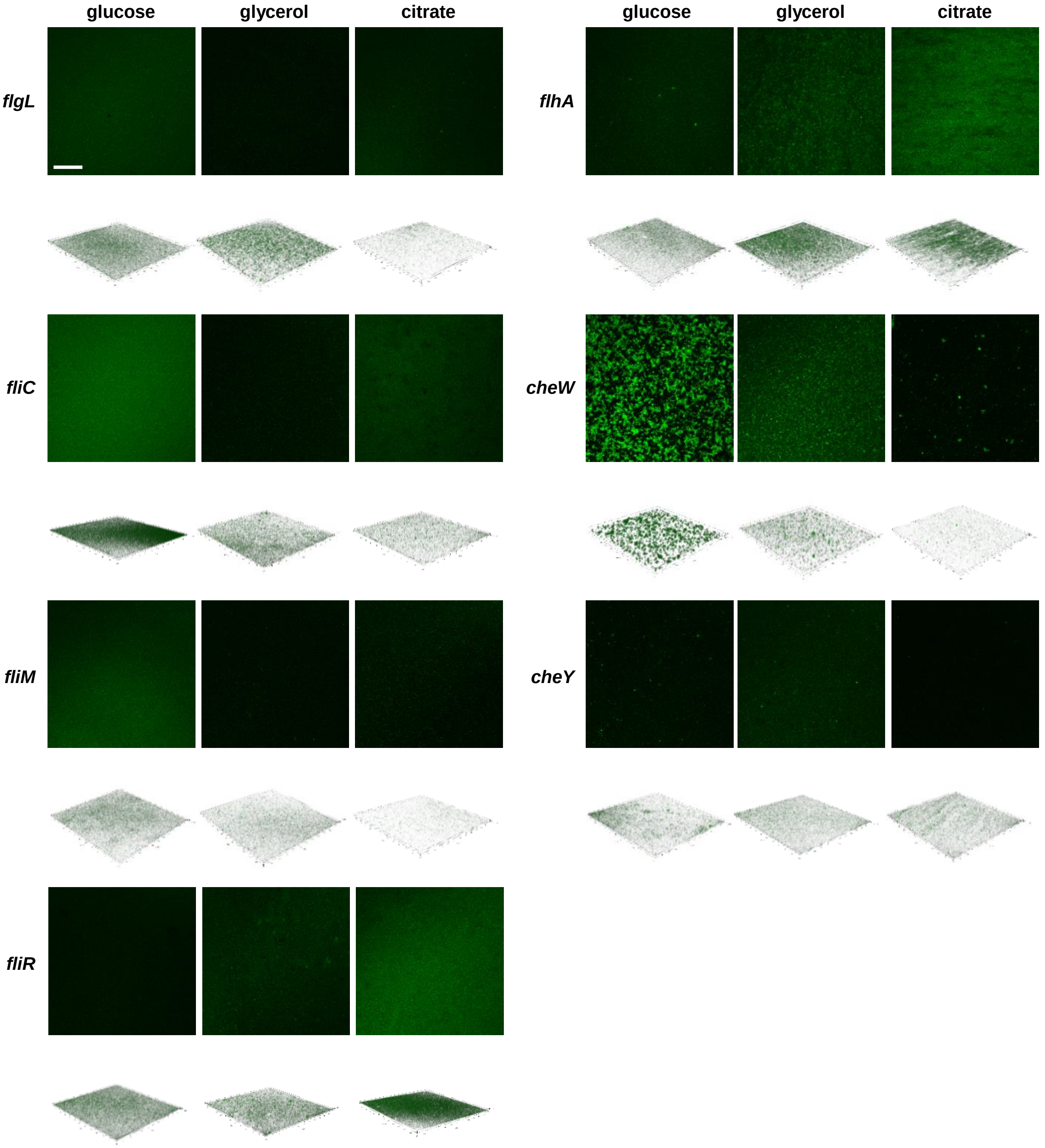

Supplementary Figure S6. Biofilm formation on glass by GFP-tagged P482 mutants in motility and attachment-related genes. Representative CLSM 2D images and 3D z-stacks of biofilm formed by GFP-tagged P482 strains on the glass bottom of 24-well plates in M9 minimal medium supplemented with 22.2 mM glucose, 43.5 mM glycerol or 20 mM citrate. Bar = 200 μm.

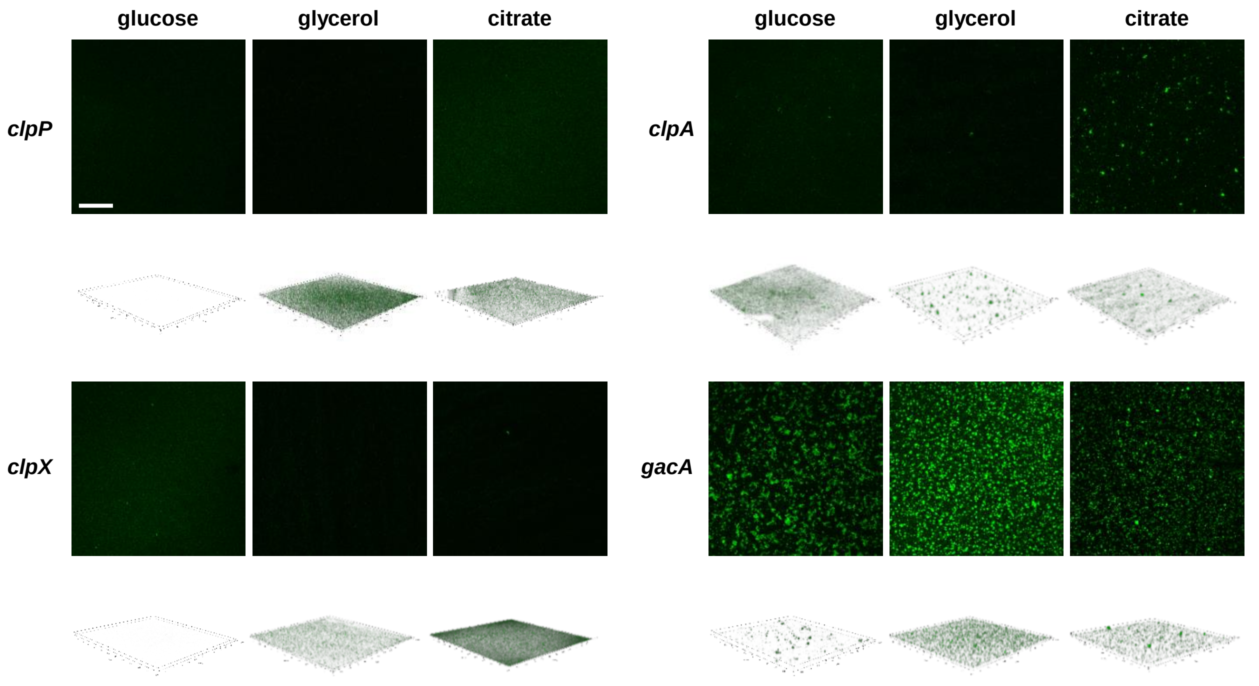

Supplementary Figure S7. Biofilm formation on glass by GFP-tagged P482 mutants in proteases-encoding and *gacA* genes. Representative CLSM 2D images and 3D z-stacks of biofilm formed by GFP-tagged P482 strains on the glass bottom of 24-well plates in M9 minimal medium supplemented with 22.2 mM glucose, 43.5 mM glycerol or 20 mM citrate. Bar = 200 µm.

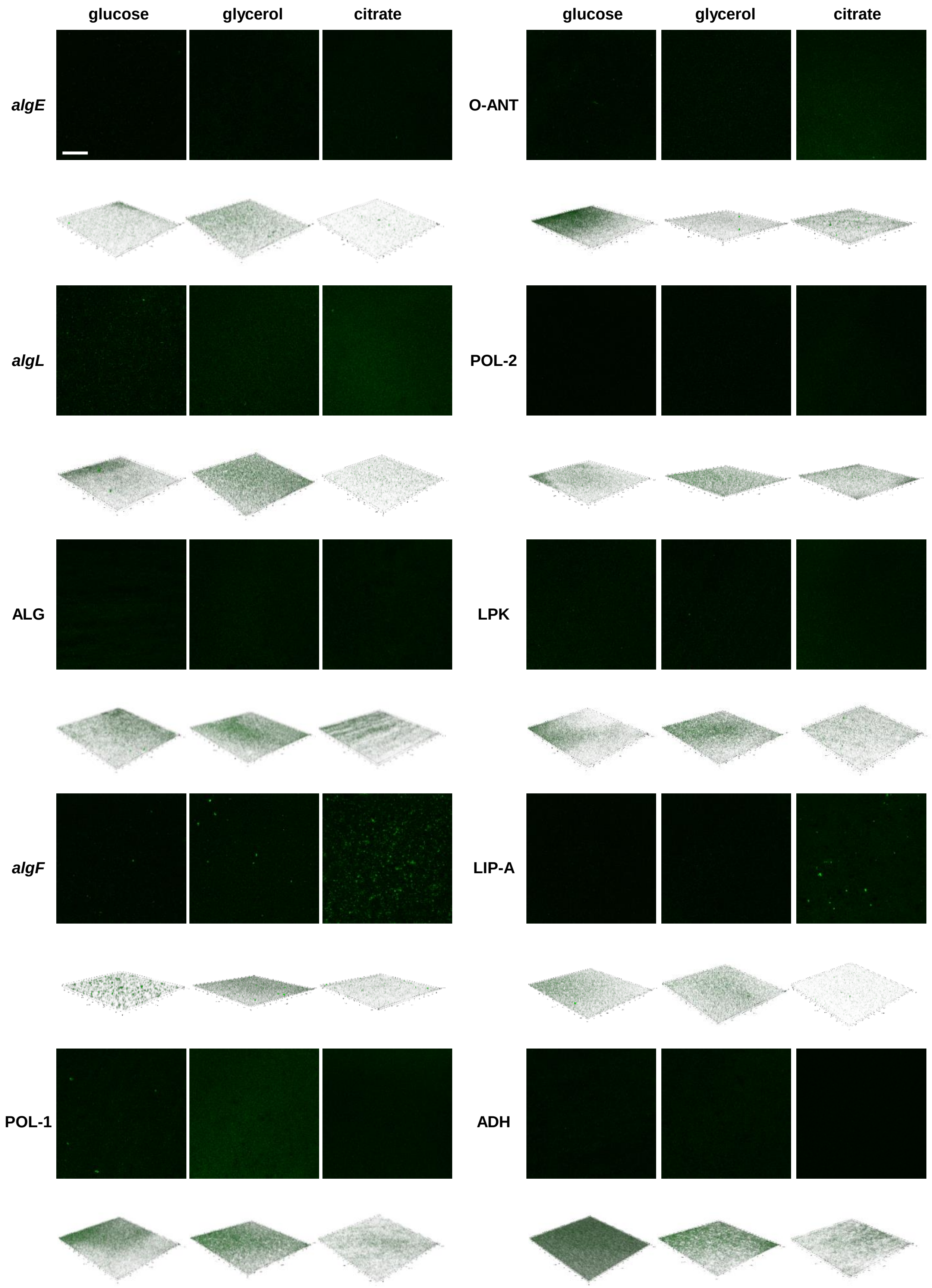

Supplementary Figure S8. Biofilm formation on glass by GFP-tagged P482 mutants in matrix synthesis-related genes. Representative CLSM 2D images and 3D z-stacks of biofilm formed by GFP-tagged P482 strains on the glass bottom of 24-well plates in M9 minimal medium supplemented with 22.2 mM glucose, 43.5 mM glycerol or 20 mM citrate. Bar = 200 µm.

Supplementary Table S1. PCR primers designed and used in this study.

**Locus**	**Name**	**Sequence**	**Introduced cloning site**	**Amplicon length (bp)**
BV82_0864	XbaI_P482_0864_F	GCGCGC**TCTAGA**CGCAGCCAGACTACGATGAC	XbaI	473
	ApaI_P482_0864_R	GCGCGC**GGGCCC**GGCGGTCTTGAACTTGATGAC	ApaI	
BV82_0868	XbaI_P482_0868_F	TTCGC**TCTAGA**CCACACCCTGGACAAGTTCT	XbaI	400
	Xho_P482_0868_R	TTAAA**CTCGAG**GAGCATGGTCAGGAACAGGT	XhoI	
BV82_0887	XbaI_P482_0887_F	GCGCGC**TCTAGA**CATGTTCAACCTGCTGCGTC	XbaI	460
	Xho_P482_0887_R	GCGCGC**CTCGAG**TCATCGAGTACGGCATGGTCAC	XhoI	
BV82_0892	XbaI_P482_0892_F	GCGCGC**TCTAGA**CGCATTCGCCTGTATTTCGCC	XbaI	481
	Kpn_P482_0892_R	GCGCGC**GGTACC**AAAGCAATGTTGACCACCAGCAG	KpnI	
BV82_0894	XbaI_P482_0894_F	GCGCGC**TCTAGA**CAACATGACCTTTGCCGATGCC	XbaI	451
	Xho_P482_0894_R	GCGCGC**CTCGAG**GAATCAGCCGATAGCCGACC	XhoI	
BV82_0850	XbaI_P482_0850_F	GCGCGC**TCTAGA**CGGCATCAACGTGTTCAAGG	XbaI	463
	Kpn_P482_0850_R	GCGCGC**GGTACC**AGTCATCGACCGTCAGCACC	KpnI	
BV82_0883	XbaI_P482_0883_F	GCGCGC**TCTAGA**ATCACAATCACCTGCTCAACGA	XbaI	542
	Kpn_P482_0883_R	GCGCGC**GGTACC**ACAGCAAGAACAACCGCTCTC	KpnI	
BV82_1101	XbaI_P482_1101_F	GCGCGC**TCTAGA**AGCGGGTGATCTTCCTGGTC	XbaI	428
	Kpn_P482_1101_R	GCGCGC**GGTACC**CAGTGTCACGCTTGATGGTCTC	KpnI	
BV82_1102	XbaI_P482_1102_F	GCGCGC**TCTAGA**GTACAACCACTACAAGCGCCT	XbaI	476
	Kpn_P482_1102_R	GCGCGC**GGTACC**GCACCGCCACAGATGAACAG	KpnI	
BV82_1251	XbaI_P482_1251_F	GCGCGC**TCTAGA**GCGGCATCTTCGAGAAAGACC	XbaI	574
	Xho_P482_1251_R	GCGCGC**CTCGAG**GAAACGCACCAGCTCAACCC	XhoI	
BV82_4694	XbaI_P482_4694_F	GCGCGC**TCTAGA**CGGCTACCGACATCATCGAGAC	XbaI	520
	Kpn_P482_4694_R	GCGCGC**GGTACC**GTTGTAGGCGTCGCTGTTGG	KpnI	
BV82_4697	XbaI_P482_4697_F	GCGCGC**TCTAGA**GATTCCACCGCGAGCATCAC	XbaI	527
	Kpn_P482_4697_R	GCGCGC**GGTACC**CGGCAGGGCATAGTTGTGGT	KpnI	

BV82_4698	XbaI_P482_4698_2_F	ATTAT**TCTAGA**ACGGTGCCAACTTCACCTAC	XbaI	445
	Xho_P482_4698_2_R	AATTC**CTCGAG**AGAATCGAGGCAACGAACAG	XhoI	
BV82_4700	XbaI_P482_4700_F	GCGCGC**TCTAGA**CCGAAAGGCTCGACCTTCGT	XbaI	392
	Kpn_P482_4700_R	GCGCGC**GGTACC**GCTGACCTTGACCGGATTGATCTC	KpnI	
BV82_5088	XbaI_P482_5088_F	GCGCGC**TCTAGA**ATCAGCCACTCGCCGATCAG	XbaI	624
	Kpn_P482_5088_R	GCGCGC**GGTACC**GCAACGAACTTGAAGTGACCACC	KpnI	
BV82_5092	XbaI_P482_5092_F	GCGCGC**TCTAGA**CGTGTTTGCCATGTTCATCGCC	XbaI	400
	Kpn_P482_5092_R	GCGCGC**GGTACC**GGATCATCTTGCTGCCGACC	KpnI	
BV82_5095	XbaI_P482_5095_F	GCGCGC**TCTAGA**ACTTGAGCATCACGTCCACGG	XbaI	476
	Xho_P482_5095_R	GCGCGC**CTCGAG**TCTTCCACATCAGTACCAGCGG	XhoI	
BV82_1850	XbaI_P482_1850_F	GCGCGC**TCTAGA**CGACTTGCACCTGGACAACCT	XbaI	492
	Kpn_P482_1850_R	GCGCGC**GGTACC**GATGTTATAGCGTTTGAGCACCAG	KpnI	
BV82_0543	XbaI_P482_0543_F	GCGCGC**TCTAGA**CGAAGCCAGGTTCTATGAAGAG	XbaI	453
	Xho_P482_0543_R	GCGCGC**CTCGAG**GAGAATCCAGTAGGTCAGCGG	XhoI	
BV82_3824	XbaI_P482_3824_F	GCGCGC**TCTAGA**CATGTTGATGATGCCCTGGT	XbaI	482
	Kpn_P482_3824_R	GCGCGC**GGTACC**TTCACAGGTCAATTCGAGGAG	KpnI	

The listed oligonucleotides were synthesized by Sigma-Aldrich (USA). Sequences recognized by respective restriction enzymes are given in bold. Annealing temperature for all primers was 65°C.

Supplementary Table S2. P values from ANOVA analyses for the listed tests for the *P. donghuensis* P482 mutants' biofilm phenotypes compared to the wild-type strain.

Comparison	Swimming	Swarming	Polystyrene (glucose)	Polystyrene (glycerol)	Polystyrene (citrate)	Glass (glucose)	Glass (glycerol)	Glass (citrate)
WT vs. *flgL*	**<0.0001**[d]	**0,0356**[a]	0,9955	**<0.0001**[d]	0,9991	**<0.0001**[d]	**<0.0001**[d]	**<0.0001**[d]
WT vs. *fliC*	**<0.0001**[d]	**0,0392**[a]	0,1385	0,6291	0,9168	**<0.0001**[d]	**<0.0001**[d]	**<0.0001**[d]
WT vs. *fliM*	**0,0004**[c]	**0,0342**[a]	**0,0175**[a]	0,3858	0,1005	0,5725	**<0.0001**[d]	**<0.0001**[d]
WT vs. *fliR*	**<0.0001**[d]	**0,0345**[a]	**0,0004**[c]	0,9654	**0,0043**[b]	0,9999	**0,0002**[c]	0,9955
WT vs. *flhA*	**<0.0001**[d]	**0,0343**[a]	**<0.0001**[d]	0,1888	**0,0002**[c]	**0,017**[a]	0,3811	0,9994
WT vs. *cheW*	0,1783	0,3357	0,0588	0,955	0,999	**<0.0001**[d]	0,9872	**<0.0001**[d]
WT vs. *cheY*	0,9943	0,9996	0,5557	0,2498	>0,9999	0,9585	**<0.0001**[d]	**<0.0001**[d]
WT vs. *clpP*	0,9023	0,9695	0,9945	0,1953	0,9883	0,2403	**<0.0001**[d]	**<0.0001**[d]
WT vs. *clpX*	0,7487	0,0538	**<0.0001**[d]	0,9957	**0,0004**[c]	0,9998	**<0.0001**[d]	**<0.0001**[d]
WT vs. *clpA*	0,9994	0,9884	0,9998	0,0715	0,9997	0,9993	**<0.0001**[d]	**<0.0001**[d]
WT vs. *gacA*	0,5542	**0,0113**[a]	**<0.0001**[d]	**<0.0001**[d]	**<0.0001**[d]	**0,0027**[b]	0,9511	**0,0111**[a]
WT vs. *algE*	0,9899	0,7851	**0,0199**[a]	0,9158	0,9995	0,7307	**<0.0001**[d]	**<0.0001**[d]
WT vs. *algL*	0,9896	0,9999	0,1164	**0,0263**[a]	>0,9999	0,9991	**<0.0001**[d]	**<0.0001**[d]
WT vs. ALG	0,9878	0,1696	**<0.0001**[d]	0,9993	0,9991	0,8166	**<0.0001**[d]	**<0.0001**[d]
WT vs. *algF*	0,9733	>0,9999	0,8542	0,9996	0,9995	0,3616	**<0.0001**[d]	**<0.0001**[d]
WT vs. POL-1	0,9996	0,0893	0,5818	**0,0049**[b]	0,9997	0,9993	**<0.0001**[d]	**<0.0001**[d]
WT vs. O-ANT	0,999	0,9991	0,1369	0,7277	0,832	0,9997	**<0.0001**[d]	**<0.0001**[d]
WT vs. POL-2	>0,9999	0,3047	0,9946	0,9949	0,9521	0,6909	**<0.0001**[d]	**<0.0001**[d]
WT vs. LPK	0,9998	0,7946	0,9248	0,1485	0,522	0,9992	**<0.0001**[d]	**<0.0001**[d]
WT vs. LIP-A	0,9956	0,9996	0,3433	0,9991	0,9994	0,9496	**<0.0001**[d]	**<0.0001**[d]
WT vs. ADH	0,9993	0,9751	0,117	0,9991	0,9993	0,0763	**<0.0001**[d]	**<0.0001**[d]

Statistically significant P values are marked in bold.

[a] – P value < 0.05, [b] - P value < 0.01, [c] – P value < 0.0005, [d] – P value < 0.0001

Supplementary Table S3. P values from ANOVA analyses comparing efficiency of biofilm formation on abiotic surfaces (polystyrene or glass) for each tested *P. donghuensis* P482 strain in the three tested carbon sources.

	**Biofilm on polystyrene**			**Biofilm on glass**		
P. donghuensis **P482 strain**	**glucose vs. glycerol**	**glucose vs. citrate**	**glycerol vs. citrate**	**glucose vs. glycerol**	**glucose vs. citrate**	**glycerol vs. citrate**
WT	**0.0002**[c]	0.0562	**<0.0001**[d]	**<0.0001**[d]	**0.0142**[a]	**0.0011**[b]
flgL	0.057	**0.0004**[c]	**<0.0001**[d]	0.0879	**0.0021**[b]	0.228
fliC	**<0.0001**[d]	**<0.0001**[d]	**<0.0001**[d]	0.2253	**0.0084**[b]	0.2551
fliM	**<0.0001**[d]	**<0.0001**[d]	**<0.0001**[d]	0.065	**0.0349**[a]	0.9513
fliR	**<0.0001**[d]	**<0.0001**[d]	**<0.0001**[d]	0.2016	**0.0205**[a]	0.4871
flhA	0.5025	**<0.0001**[d]	**<0.0001**[d]	0.1446	0.85	0.3469
cheW	**<0.0001**[d]	**0.0005**[c]	**<0.0001**[d]	0.8393	**<0.0001**[d]	**<0.0001**[d]
cheY	**<0.0001**[d]	**<0.0001**[d]	**<0.0001**[d]	**0.0478**[a]	0.1169	**0.0004**[c]
clpP	**<0.0001**[d]	0.1776	**<0.0001**[d]	**<0.0001**[d]	0.2992	**<0.0001**[d]
clpX	**0.0008**[c]	**0.0013**[b]	**<0.0001**[d]	**<0.0001**[d]	**<0.0001**[d]	0.2158
clpA	**<0.0001**[d]	**0.0352**[a]	**<0.0001**[d]	0.1386	**0.0018**[b]	**<0.0001**[d]
gacA	**<0.0001**[d]	>0.9999	**<0.0001**[d]	0.1521	0.6253	**0.0236**[a]
algE	**0.0001**[c]	**<0.0001**[d]	**<0.0001**[d]	0.7781	0.2681	0.0813
algL	**<0.0001**[d]	**0.0264**[a]	**<0.0001**[d]	0.8176	0.057	0.1785
ALG	**<0.0001**[d]	**<0.0001**[d]	**<0.0001**[d]	**0.0002**[c]	0.8742	**<0.0001**[d]
algF	**<0.0001**[d]	**0.0271a**	**<0.0001**[d]	**<0.0001**[d]	**<0.0001**[d]	0.1307
POL-1	**<0.0001**[d]	0.055	**<0.0001**[d]	0.2147	0.1724	**0.0054**[b]
O-ANT	**<0.0001**[d]	**<0.0001**[d]	**<0.0001**[d]	**0.0026**[b]	**<0.0001**[d]	0.2214
POL-2	**<0.0001**[d]	**0.0348**[a]	**<0.0001**[d]	0.4445	0.077	0.5444
LPK	**<0.0001**[d]	**0.0076**[b]	**<0.0001**[d]	**0.026**[a]	0.7468	**0.005**[b]
LIP-A	**<0.0001**[d]	**0.0211**[a]	**<0.0001**[d]	0.5378	0.1996	0.7633
ADH	**<0.0001**[d]	**<0.0001**[d]	**<0.0001**[d]	**<0.0001**[d]	0.4834	**<0.0001**[d]

Statistically significant P values are marked in bold.

[a] – P value < 0.05, [b] - P value < 0.01, [c] – P value < 0.0005, [d] – P value < 0.0001

Supplementary Table S4. Mean values of biofilm thickness measurements on glass, with SEM values and P values from ANOVA analyses comparing efficiency of biofilm formation on the abiotic surface for each tested *P. donghuensis* P482 strain in the three tested carbon sources.

	**Biofilm thickness parameters – mean (SEM) [µm]**			**P values for each strain vs WT**		
***P. donghuensis* P482 strain**	**glucose**	**glycerol**	**citrate**	**glucose**	**glycerol**	**citrate**
WT	44.95 (3.85)	46.58 (5.86)	34.76 (2.33)	-	-	-
flgL	22.28 (1.97)	21.66 (1.67)	19.09 (2.20)	**<0.0001[d]**	**<0.0001[d]**	**<0.0001[d]**
fliC	24.14 (3.61)	16.25 (0)	17.46 (2.44)	**<0.0001[d]**	**<0.0001[d]**	**<0.0001[d]**
fliM	15.78 (0.61)	18.41 (1.67)	17.87 (0.86)	**<0.0001[d]**	**<0.0001[d]**	**<0.0001[d]**
fliR	18.57 (1.80)	22.20 (1.59)	22.75 (3.47)	**<0.0001[d]**	**<0.0001[d]**	**<0.0001[d]**
flhA	20.42 (0.79)	33.27 (4.43)	19.90 (2.02)	**<0.0001[d]**	**<0.0001[d]**	**<0.0001[d]**
cheW	58.03 (2.18)	34.12 (3.81)	24.14 (2.94)	**<0.0001[d]**	**0,0074[b]**	**0,0002[c]**
cheY	26.46 (1.73)	23.46 (4.47)	21.12 (0.86)	**<0.0001[d]**	**<0.0001[d]**	**<0.0001[d]**
clpP	19.5 (2.09)	17.87 (0.89)	15.75 (5.16)	**<0.0001[d]**	**<0.0001[d]**	**<0.0001[d]**
clpX	24.14 (2.79)	14.08 (1.67)	17.87 (6.08)	**<0.0001[d]**	**<0.0001[d]**	**<0.0001[d]**
clpA	27.44 (2.75)	35.75 (1.78)	24.37 (0.86)	**<0.0001[d]**	**0,0124[a]**	**0,0001[c]**
gacA	50.60 (4.67)	42.25 (6.33)	36.96 (4.06)	0,8974	0,0764	0,9951
algE	22.28 (1.73)	17.87 (0.89)	16.25 (1.73)	**<0.0001[d]**	**<0.0001[d]**	**<0.0001[d]**
algL	26.46 (2.88)	24.37 (3.20)	18.68 (1.44)	**<0.0001[d]**	**<0.0001[d]**	**<0.0001[d]**
ALG	23.21 (1.73)	22.62 (2.61)	21.12 (1.50)	**<0.0001[d]**	**<0.0001[d]**	**<0.0001[d]**
algF	48.75 (7.90)	16.79 (2.15)	16.25 (2.74)	0,9994	**<0.0001[d]**	**<0.0001[d]**
POL-1	26.46 (3.17)	21.58 (0.79)	23.56 (1.14)	**<0.0001[d]**	**<0.0001[d]**	**<0.0001[d]**
O-ANT	25.07 (1.80)	15.16 (0.83)	16.25 (1.73)	**<0.0001[d]**	**<0.0001[d]**	**<0.0001[d]**
POL-2	19.96 (3.17)	11.91 (0.83)	17.87 (4.34)	**<0.0001[d]**	**<0.0001[d]**	**<0.0001[d]**
LPK	21.35 (0.86)	25.87 (3.94)	25.59 (2.20)	**<0.0001[d]**	**<0.0001[d]**	**0,0013[b]**
LIP-A	19.5 (4.69)	17.33 (1.32)	17.06 (1.14)	**<0.0001[d]**	**<0.0001[d]**	**<0.0001[d]**
ADH	26 (0)	16.78 (0.41)	24.37 (0.86)	**<0.0001[d]**	**<0.0001[d]**	**0,0001[c]**

Statistically significant P values are marked in bold.

[a] – P value < 0.05, [b] - P value < 0.01, [c] – P value < 0.0005, [d] – P value < 0.0001

Supplementary Table S5. Comparison of biofilm formation efficiencies on polystyrene and glass for each tested *P. donghuensis* P482 strain in the three tested carbon sources.

	**Biofilm on polystyrene**			**Biofilm on glass**		
***P. donghuensis* P482 strain**	**glucose**	**glycerol**	**citrate**	**glucose**	**glycerol**	**citrate**
flgL vs. WT	***	*	***	****	*	*
fliC vs. WT	****	**	***	****	*	*
fliM vs. WT	****	**	****	***	*	*
fliR vs. WT	****	**	****	***	**	***
flhA vs. WT	****	**	****	****	***	***
cheW vs. WT	****	***	***	*****	***	**
cheY vs. WT	***	**	***	**	*	*
clpP vs. WT	***	***	***	**	*	*
clpX vs. WT	****	***	****	***	*	*
clpA vs. WT	***	***	***	***	*	*
gacA vs. WT	*****	****	*****	****	***	**
algE vs. WT	****	***	***	**	*	*
algL vs. WT	****	***	***	***	*	*
ALG *vs.* WT	*****	***	***	**	*	*
algF vs. WT	***	***	***	***	*	*
POL-1 *vs.* WT	****	****	***	***	*	*
O-ANT *vs.* WT	****	***	***	***	*	*
POL-2 *vs.* WT	***	***	**	**	*	*
LPK *vs.* WT	***	***	***	***	*	*
LIP-A *vs.* WT	****	***	***	**	*	*
ADH *vs.* WT	****	***	***	*	*	*

* - biofilm formation efficiency below 50% of WT; ** - biofilm formation efficiency below 100% of WT; *** - biofilm formation efficiency comparable to WT (100%); **** - biofilm formation efficiency ca. 2-fold of WT; ***** - biofilm formation efficiency ca. or over 3-fold of WT
